# Development, fabrication, and applications of laser-induced graphene-based biosensors in food and dairy sectors

**DOI:** 10.1007/s00604-025-07395-4

**Published:** 2025-08-04

**Authors:** Muhammad Ikram, Prafulla Salunke, Arshid Numan, Mazhar Sher

**Affiliations:** 1 Department of Agricultural and Biosystems Engineering, South Dakota State University, Brookings, SD 57007 USA; 2 Dairy and Food Science Department, Midwest Dairy Foods Research Center, South Dakota State University, Brookings, SD 57007 USA; 3 Sunway Centre for Electrochemical Energy and Sustainable Technology (SCEEST), Faculty of Engineering and Technology, Sunway University, Selangor Darul Ehsan, 47500 Bandar Sunway, Malaysia

**Keywords:** Laser-induced graphene, Biosensor fabrication, Sensing mechanism, Biosensors, Food and dairy

## Abstract

Laser-induced graphene (LIG) has emerged as a cutting-edge carbon material with a unique porous architecture and superior electrochemical properties. Owing to its promising potential to immobilize various biological analytes, LIG has gained intense interest in the development of next-generation biosensors. Direct laser scribing on natural or polymeric substrate materials produces LIG electrodes with tunable properties, offers controlled microstructures, ease surface modifications, and doping with suitable elements, making it promising for electroanalytical measurements. Furthermore, LIG technology stands out as being cost-effective and supports environmental sustainability and eco-conscious solutions. These diverse features open new frontiers, making it suitable for fundamental applications in diverse fields, particularly in the food and dairy industry, where rapid, on-site, and precise monitoring is vital. This review comprehensively discusses fabrications of LIG-based biosensors with a focus on various laser sources, substrate materials, and surface modifications. The core sensing mechanisms of LIG biosensors are thoroughly summarized which enable high sensitivity and selectivity. However, special attention is given to LIG biosensors’ applications in the food and dairy industry for the monitoring of food pathogens, food ingredients, food spoilage, biogenic amines, food additives, antibiotics, chemical contaminants, and pesticides. Finally, this review discusses the current challenges of LIG-based biosensors, such as reproducibility, stability, and integration into commercial industries, while offering a future outlook for potential applications. By highlighting recent advances and summarizing knowledge gaps, this review provides new insights into LIG-based sensors and their applications in the food and dairy industry to ensure food quality and safety.

**Graphical Abstract:**

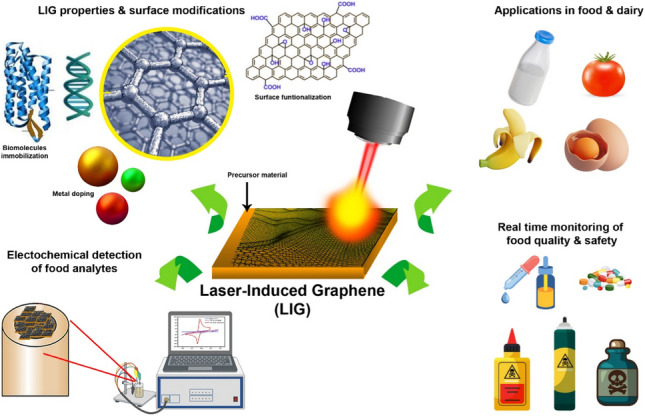

## Introduction

Observing the food and dairy product status and their hygienic packaging are key elements to prevent food spoilage because it impacts around 600 million people worldwide and causes the death of approximately 420,000 people yearly [1, 2]. Hence, adequate food and dairy product monitoring is mandatory and requires particular prerequisites. In the era of biosensing, graphene-made sensors are promising contenders in the food and dairy industry. Owing to the unique properties of graphene, it is considered a highly foreseen biosensor for food and dairy products. In addition, current advancements in LIG-derived materials have effectively broadened their spectrum in various applications. The multifunctional LIG-based biosensor developments are noteworthy and enable the real-time monitoring of physiological biomarkers, nutrients, biogenic amines, microbial contaminations, pathogens, fouling biofilms, and antibiotic concentration for the assessment of food and dairy product quality [3–5]. The progress in the production of LIG-based sensors reflects their potential to address the challenges in the modern food and dairy industry [6–8].

Graphene has gained huge attention after its discovery and widely studied two-dimensional (2D) nanostructure with some promising and commercially available products. Its unique rheological, physical, and electrochemical attributes make it a suitable material for various applications, including energy storage, electrocatalysis, flexible electronics, and biosensors [9–12]. Graphene is just one atom sheet having sp^2^ electronic configurations of carbon atoms with a honeycomb lattice, sharing sigma bond (σ) in the plane, and pi (π) bond out of plane with neighboring atoms with about 1.42 Å interatomic distance [13–15]. Graphene has a higher surface area-to-volume ratio (~ 2630 m^2^/g), higher conductivity (~ 10⁶ to 10⁷ S/m), carrier mobility, excellent mechanical attributes, greater stability, and good biocompatibility. These properties make graphene a promising and interesting candidate for electrochemical applications, including biosensing, energy storage, catalysis, electronic, environmental remediation, and other biomedical applications [16–18].

In the literature, multiple graphene synthesis techniques have been reported, encompassing thermal decomposition, hydrothermal, precipitation, chemical vapor deposition, reduction, and mechanical exfoliation [19, 20]. However, these developing strategies have certain limitations, including high processing cost, high temperature, complex synthesis routes, expensive precursors and capping agents, and poor-quality graphene, which limits their commercial applications. To cope with these challenges, intensive efforts have been made to develop a chemical-free, cost-effective, environment-friendly, and low-temperature method to attain high-quality graphene and graphene-derived materials. Hence, for the commercial application of graphene, simple and cost-effective methods are still in high demand [21–23].

Laser-induced graphene (LIG) was first reported by Lin et al. [24]. Their research presented laser scribing strategy in which laser irradiation transforms the polymeric substances into graphene by inducing photo-thermal and chemical conversion during the fabrication process; the resulting graphene is commonly known as laser-induced graphene (LIG) [24]. Laser-induced technology is extensively applied in designing multiple sensing materials and has succeeded in reducing graphene oxide into graphene. Laser irradiation is a simple and direct process to convert polymeric substances, including polyimide, polytetrafluoroethylene, and natural precursors encompassing food, wood, and lignin, into two-dimensional and three-dimensional graphene [25–29]. The laser-induced graphene synthesis is cost-effective and environmentally friendly, in which carbon dioxide (CO_2_) infrared laser converts sp^3^ hybridized carbon (C) into sp^2^ hybridized carbon by photo-thermal or photo-chemical conversion, resulting in graphene having greater surface area to volume ratio, higher thermal stability as well as electronic conductivity [25, 30]. These attributes make LIG an interesting and promising material for biosensors. LIG has been extensively studied and applied in different sensors on electrochemical, piezo resistivity, and surface acoustic waves [31–33]. LIG can be performed at ambient temperature by applying a laser directly to a precursor substance. This technology has advantages over the conventional methods applied in synthesizing graphene, including bottom-up approaches. Furthermore, LIG technology is facile and cost-effective with minimal e-waste and supports sustainable economic strategies, and can be applied directly for the printing of biosensor devices with desired patterns. Hence, the laser-induced graphene strategy is a cutting-edge technology and can be effectively applied to develop graphene-based biosensors [25, 34–36].

Despite the growing interest in LIG-based electrochemical biosensors, their fundamental applications in the food and dairy industry still remain unexplored, with key gaps in the literature, food safety applications including the detection of food pathogens, contaminants, adulterants, and antibiotics. The current review provides a brief overview of LIG-based biosensors development and their applications in food and dairy products. We described comprehensively sensing mechanisms, fabrication processes, and materials, and elaborated on their current decade breakthrough for the real-time monitoring of food and dairy product quality. Finally, a short analysis of current challenges and future perspectives gives new insights for further ongoing research and the evolution of laser-induced graphene-based biosensors for realm assessment of food and dairy product quality. This review provides key insights into the potential of LIG biosensors to revolutionize the food and dairy industry by food safety monitoring through rapid, cost-effective, and on-site detection technologies.

Recently, several articles have been published presenting the innovations regarding LIG techniques and graphene-based sensors. For instance, Zhang et al. [37] summarized the work of electrochemical laser-induced graphene sensors for medical diagnosis, and Kumar et al. [38] studied how LIG could be synthesized and its sensing mechanism in the environment. Similarly, some other articles have summarized LIG-based biosensors for food safety applications. But all these research articles lack knowledge about the development of sensors based on laser-induced graphene applicable in the food and dairy industries. This review article presents an exclusive study on LIG biosensors, explaining laser parameters, chemistry of substrate, mode of sensing, modifications relating to surface, and use in **real-world scenarios**, presenting additional integration specific to **the** food industry perspective. The key contribution of this article is its focus on all previously identified gaps present in current research.

## Fabrications of LIG-based biosensors

Graphene can be developed effectively by laser irradiation followed by patterned pathways under controlled conditions. The patterning and development of graphene are concurrently produced, in addition to traditional fabrication methods such as photolithography, which involves a complex formation encompassing several steps. Patterned LIG development under laser exposure is based on photothermal or photochemical effects. It is suggested that after infra-red laser irradiation, the bond as well as chain break occurred due to the photothermal effect. On the other hand, under UV laser irradiation, the development mechanism can be based on photochemical effects; photon energy and chemical bond energy are equivalent, leading towards the break of chemical bonds in organic substrates. However, it is also assumed that the photothermal effect can be involved in UV laser irradiation. Development of LIG under the exposure of visible light lasers is mainly based on both photothermal and photochemical effects [39–42].

**Fig. 1:**
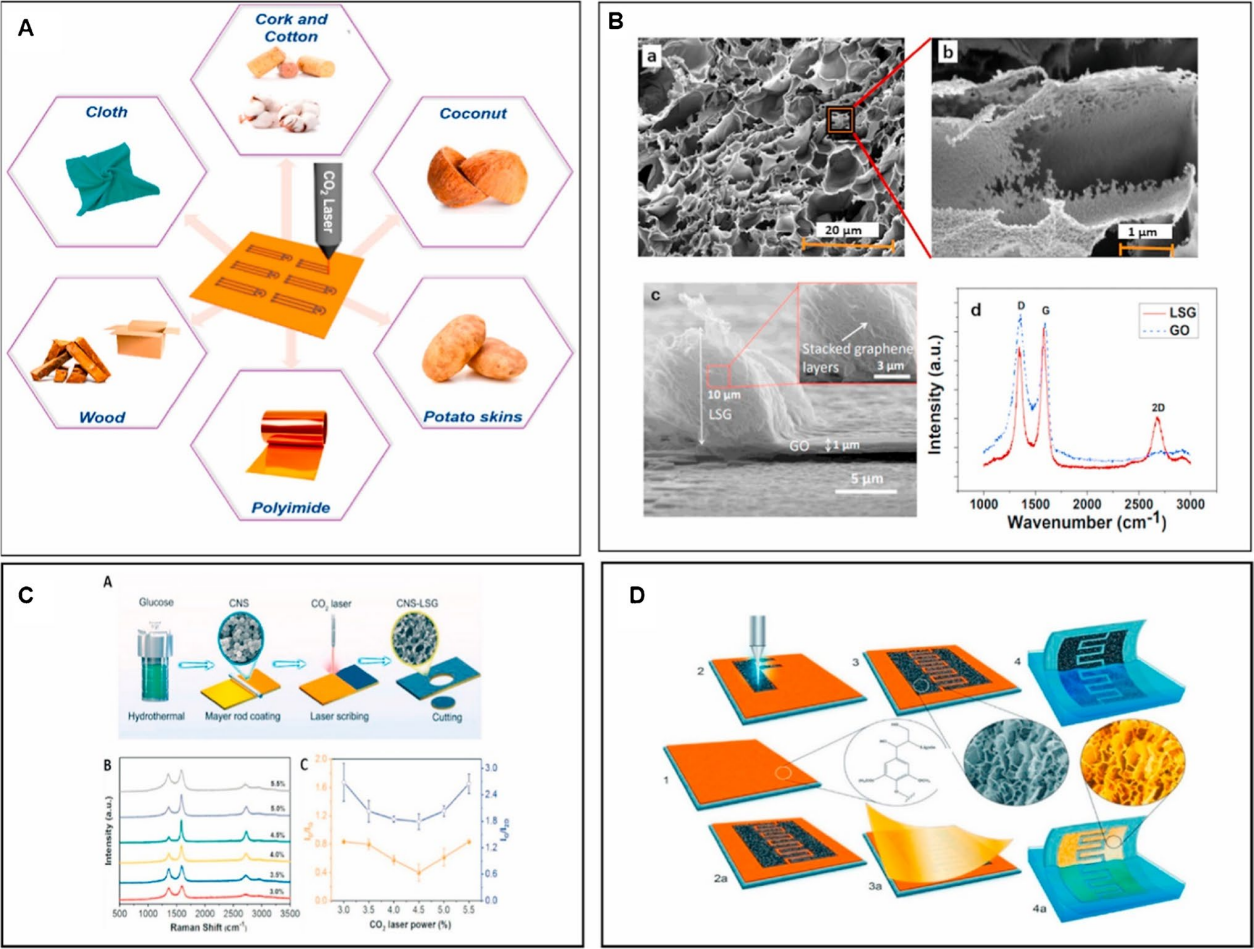
**A** Schematic illustration of effective substrates used in LIG fabrication. **B** SEM images of laser-derived graphene (LDG) at (a) 20 μm, (b) 1 μm, (c) cross-sectional SEM image, and (d) Raman spectra of fabricated LDG. **C** Shows (a) Laser scribed graphene fabrication by laser scribing, (b) Raman spectra of designed carbon nanosphere-laser scribed graphene at different laser powers, and (c) ID/IG and IG/I2D peak ratio of LDG. **D** Depicts a 3D demonstration of laser derived graphene (LDG) development by laser scribing on lignin/PVA film deposited on polyimide sheet and gold coating of fabricated LDG. Reproduced with permission under copyright © 2020 Elsevier B.V. [48]

In the current decade, LIG has gained huge attention in graphene research. Hence, it is mandatory to comprehend the fabrication mechanisms of graphene to develop biosensor devices applied in food and dairy products. The fabrication process of graphene is based on the attributes of laser source and carbon precursors. Furthermore, the exposure time and wavelength of the laser pulse are also key elements. Mostly, laser produces a photo-thermal effect in which the precursor substance absorbs the photon energy and transforms it into thermal energy, resulting in high temperature and induced carbonization, graphitization as well, and exfoliation mechanisms to develop LIG. Furthermore, during this process, the production of gaseous products generates a high pressure that makes a vital contribution to the development of porous LIG structures. In addition, laser pulses for ultrashort duration can generate nonlinear interactions with precursor substances to develop a random pattern of graphene under appropriate conditions (Fig. 1) [43–46]. CO_2_ lasers have been extensively used due to specific characteristics such as accessibility, ease of operation, and cost-effectiveness. It is mandatory to optimize the fabrication process considering the consistency and specific needs of application [47].

### Lasers for LIG biosensors fabrication

**Fig. 2:**
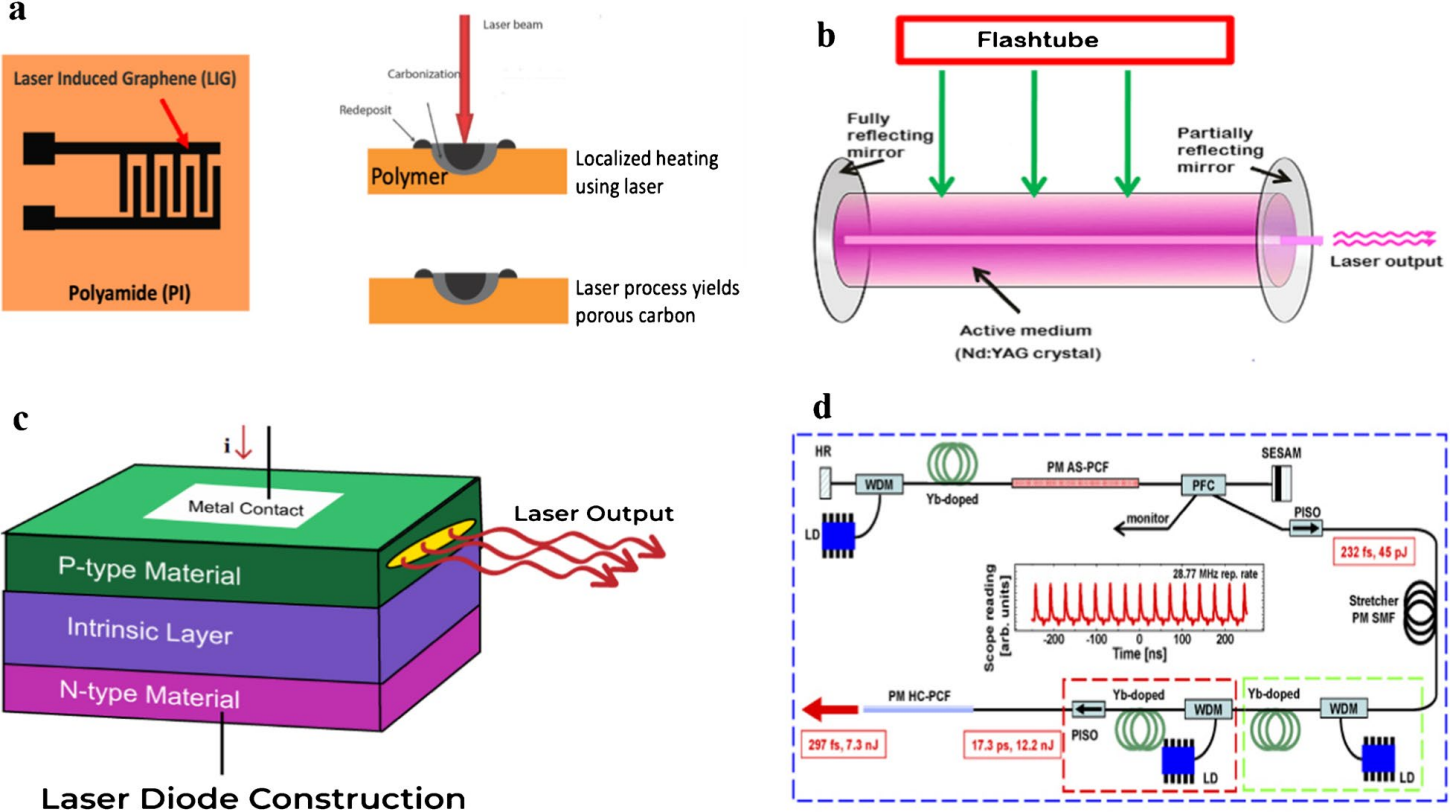
**a** LIG development by laser irradiation on a polymeric substrate. **b** Nd:YAG crystal laser for LIG fabrication. **c** Schematic illustration of semiconductor laser diode construction. **d** Femtosecond Yb-fiber laser system based on photonic crystal fibers for LIG fabrication. Adapted with permission from OPTICA under copyright CC BY 4.0 [51].

Various types of lasers with different features and wavelengths are being used in the development of LIG biosensors. These lasers include CO_2_ lasers, semiconductor diode lasers, fiber lasers, and neodymium doped yttrium garnet (Nd: YAG) Lasers. The mechanism of laser fabrication depends on laser wavelengths, exposure time, power, and scanning speed. The varying laser wavelength generates unique photothermal and photochemical effects, hence modulating the LIG sensor surface porosity, area, conductivity, and types of functional groups. On the other hand, laser operation modes encompassing continuous and pulsed lasers also have key impact on the LIG sensors development and properties. Continuous lasers are proposed for the fabrication of large surface areas, while pulsed lasers are proposed for smaller areas due to high energy density. The development of LIG sensors starts with the selection of suitable and carbon-rich substrates such as PI sheet, which is then subjected to laser treatment. The laser induction on substrate materials generates a photochemical or photothermal effect to convert sp^3^ carbon into sp^2^ carbon [48–50]. Figure 2 shows the schematic illustration of the LIG biosensors fabrication.

#### CO_2_ lasers

In the current era, infrared CO_2_ lasers are extensively applied to fabricate LIG-based biosensors. In comparison to previously reported continuous wave lasers, CO_2_ lasers have greater efficiency, including higher output power between 0.1 and 60 kW, enhanced energy in the range of 10–30%, excellent reliability, and lifetime. CO_2_ lasers have a low processing cost at the commercial scale among all available lasers, making them highly promising and applicable for industrial-scale production. Furthermore, the emission spectra of CO_2_ lasers have a 9–11 µm range, which is effectively absorbed by the C–C bonding in precursor substance. In addition, CO_2_ lasers have the potential to run in pulse mode besides continuous mode with varying repetition rates between a few Hz and more than 100 kHz, resulting in tunable and excellent attributes for the development of biosensors for various applications in food and dairy products. In LIG synthesis CO_2_ laser creates direct writing on the PI sheet, converting carbon atoms into graphene via thermal effect. The whole process is performed at ambient conditions without vacuum or inert gas environment. The LIG generated by this rapid method has higher scalability, is cost-effective, and is environmentally friendly. However, there are limitations of CO_2_ lasers, including low resolution of spatial patterns due to long-wavelength emission spectra, such as the minimum spot size having a range of 60–120 µm [47, 52–54].

#### Neodymium doped yttrium garnet (Nd: YAG) lasers

To overcome abovementioned limitations, neodymium-doped yttrium garnet (Nd: YAG) solid-state lasers have been suggested to have an emission spectrum of 1064 nm. They have applied CO_2_ lasers to produce LIG-based biosensors. Their working principle is based on continuous as well as pulse modes. The YAG crystals have the capability to generate higher power density at the ultra-short duration of laser pulse exposure. In addition, the emitted light from the Nd: YAG laser source can penetrate through different optical fibers, enabling the formation of compact systems. Both continuous wave and pulsed mode Nd: YAG lasers have been effectively applied to fabricate LIG biosensors. However, the resistance was not up to par compared to CO_2_ lasers. Furthermore, Nd: YAG lasers have certain limitations, including lower power, inefficient beam quality, and poor energy efficiency, which limit their applications in LIG biosensors fabrications. To cope with these challenges, neodymium-doped yttrium orthovanadate (Nd: YVO4) lasers have been suggested as a replacement for Nd: YAG, accompanied by their greater energy efficiency, minimum heat production, excellent lifespans, and varying wavelengths. These attributes make them highly applicable and reliable laser sources for the fabrication of LIG derived from polymers and cork [55, 56].

#### Semiconductor diode-based lasers

For the replacement of CO_2_ and Nd: YAG lasers to overcome their limitations, semiconductor diode lasers have been reported to fabricate LIG-based biosensors. Diode lasers have certain advantages, including higher efficiency, greater reliability, cost-effectiveness, light weight, compact size, and varying wavelengths in the 405–3330 nm range. Furthermore, diode lasers have excellent electrochemical and tunable optical properties, resulting in greater power transform efficiency of up to 50%. In addition, diode lasers are commercially available with output power in the range of a few milliwatts to kilowatts. Hence, they are replacing other laser sources for multiple applications. The low-power diode lasers are more promising as compared to high-power diode lasers, because they have good beam and low beam quality, respectively. Diode lasers with wavelengths in the visible region are recommended for the fabrication of LIG-based biosensors. Diode lasers are cost-effective, enhance spatial resolution, and have greater absorption of precursor substances. These properties make them interesting and promising for LIG fabrications [57–59].

#### Fiber lasers

In the current decade, fiber lasers have been widely used in the fabrication of LIG-based biosensors. Fiber lasers’ working mechanism is based on optical fibers modified with rare earth elements comprising erbium and ytterbium. Fiber lasers are highly efficient because the light generated from optical fibers has enhanced optical gain, accompanying effective pump-signal interaction, resulting in greater output power of about multi kilowatt. Furthermore, fiber lasers have excellent thermal management because of a higher surface area to volume ratio, enhanced stability, good beam quality, easy maintenance, and small size footprints [60, 61]. The ytterbium (Yb) modified fiber lasers have gained huge attention owing to their greater quantum efficiency of about 94%. In addition, Yb modified fiber lasers, having mode-locked, can generate ultra-short pulses of picoseconds or even femtoseconds. The femtosecond fiber lasers can produce a repetitive pulse in the range of 1020–1070 nm wavelength with a pulse repetition rate of a few tens to about hundreds of MHz. The power intensity of the pulse peak approached up to megawatts. The ultra-short pulse exposure time minimizes the heat to a greater extent in comparison to longer pulse duration or continuous wave mode lasers. The enhanced peak power intensity makes nonlinear absorption to transparent materials possible under particular laser wavelengths. Moreover, nonlinear absorption is confined to a particular area or volume to attain high-resolution patterning. Hence, fiber lasers have enhanced spatial resolution, potential for direct patterning, and reduced photo-thermal ablation properties, which make them versatile light sources for LIG-based biosensor fabrication [62–64].

## Materials for LIG biosensors fabrication

The performance and quality of LIG biosensors are dependent on the selection of substrate materials and laser parameters. A broad range of materials has been reported, including polymers and natural substances that can be used as carbonaceous predecessors. However, the use of renewable and recyclable substrate materials like green sources is highly recommended for LIG fabrication. These materials present a sustainable and environmentally friendly alternative to synthetic polymers. Besides substrate materials, the laser length, pulsing speed, control conditions, and chemical and structural composition of carbonaceous predecessors are major factors in predicting whether the desired carbon precursors can be transformed into LIG. A wide range of carbon materials have been suggested for LIG doping for the desired chemical and physical attributes enlisted in Table 1 [65–68]. Table 1 presents fabrication approaches of LIG sensors, illustrating the variety of lasers, nature of substrate material, and the modification process. Scalability and cost-efficiency bring domination of CO_2_ lasers, whereas other types of lasers enhance spatial resolution. The influence of fabrication parameters on conductivity could be observed via sheet resistance.

**Table 1:** LIG-based biosensor fabrications, materials, and laser types

Fabrication methods	Laser type	Fabrication/modification materials	Substrate materials	Sheet resistance	Reference
Direct laser writing	CO_2_ laser	Copper nanocubes	Polyimide	14.3 Ω cm^−2^	[69]
Direct laser writing	X-660 laser cutter	Aptamer	Polyimide	-	[70]
Direct laser writing	VLS 3.50 laser cutter	PEDOT	Polyimide	-	[71]
Direct laser writing	CO_2_ laser	Antibodies	Polyimide	18.2 MΩ	[72]
Laser engraving	CO_2_ laser	AuNPs	Polyimide	0.47 ± 0.03 kΩ	[73]
Laser scribing and inkjet printing	CO_2_ laser	Antibodies	Polyester	90.6 ± 3.9 Ω/sq	[74]
Direct laser writing	CO_2_ laser	-	Cellulose paper	43.7 ± 2.3 Ω sq^−1^	[75]
Laser scribing	CO_2_ laser	Silicone	Polyimide	10.96 Ω cm^−2^	[71]
Laser scribing	CO_2_ laser	PtAuNP	Polyimide	43 Ω cm^−2^	[65]
Laser scribing	VLS 3.50 laser cutter	Erichrome black T/amine/ PEDOT	Polyimide	102.4 ± 7.3 Ω cm^−2^	[71]
Laser scribing	CO_2_ laser	MIP/PPy	Polyimide	58 Ω cm^−2^	[30]
Laser engraving	CO_2_ laser	Cu^2+^/ethyl-cellulose	Polyimide	75.5 Ω	[76]
Laser engraving	Ultraviolet marking machine	AgNPs	Polyimide	-	[77]
Laser ablation	CO_2_ laser	AuNPs-pPy-chitosan	Polyimide	35 ± 3 Ω	[78]
Laser scribing	CO_2_ laser	nanoPd@LIG	Polyimide	-	[79]
Laser engraving	CO_2_ laser	Tetraphenylporphyr-in/ZnO	Polyimide	-	[80]
Laser ablation	Fiber laser	Cu-LIG/ITO	Phenolic film	110 Ω,	[81]
Direct laser writing	CO_2_ laser	Cellulose paper	Polyimide	105 Ω sq^−1^	[82]
Direct laser writing	CO_2_ laser	-	Cork	10.6 Ω sq^−1^	[83]
Direct imprinting	Femtosecond laser	-	GO films	3.91 × 10^−5^ Ωm	[84]
Direct laser writing	CO_2_ laser	PDMS-TEG	PDMS	130 Ω sq^−1^	[85]
Direct laser writing	Semiconductor laser	PR-Fe coating	Phenolic resin	44 Ω sq^−1^	[86]

### Polymeric Materials

**Fig. 3:**
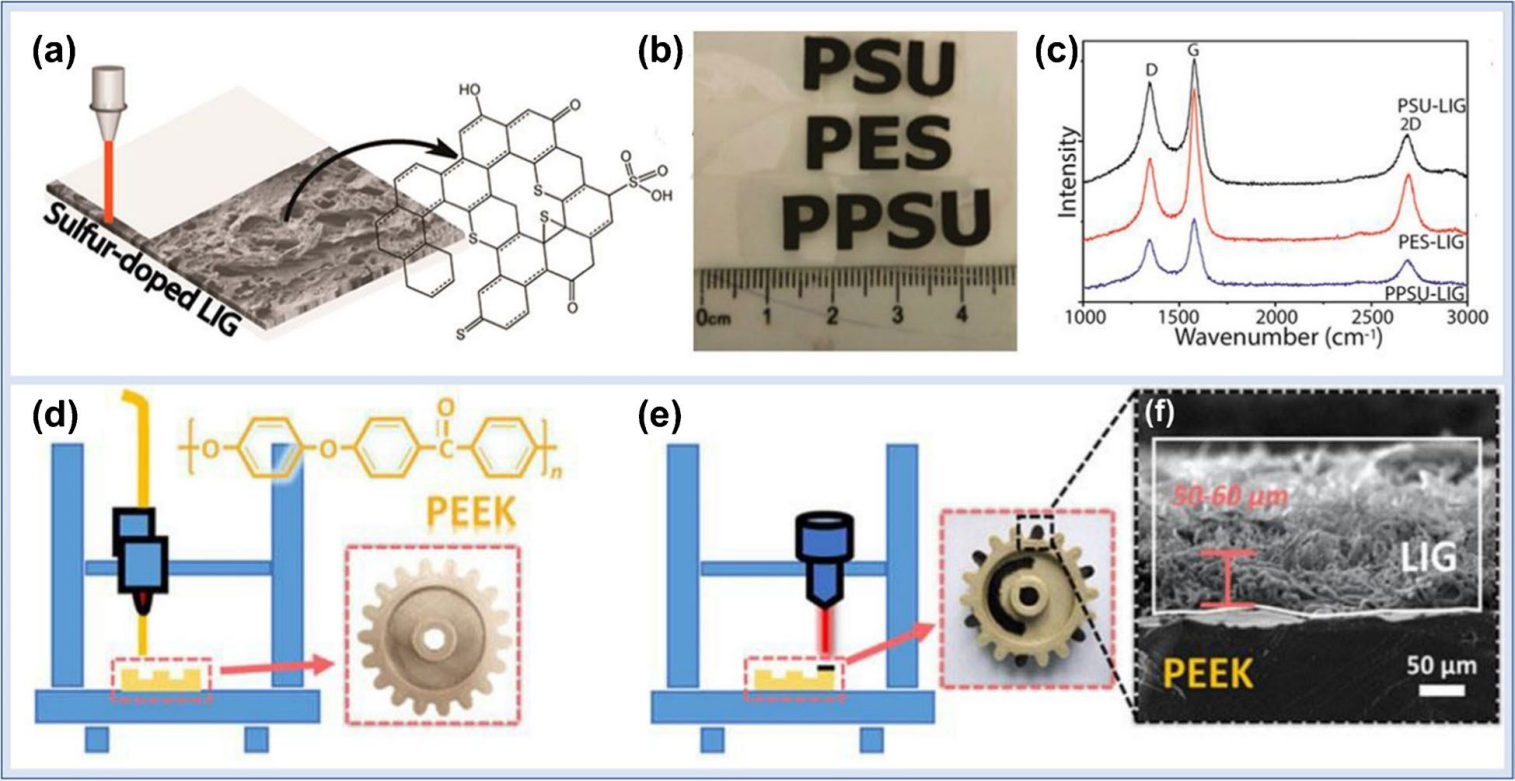
Schematic illustration of LIG fabrication. **a** Development of LIG on a polysulfone precursor material. **b** Image of LIG patterned on different polymer substrates. **c** Raman spectra of LIG developed using various polymeric materials. **d** PEEK components development via 3D printing. **e** Development of LIG on PEEK substrate. **f** SEM images of fabricated LIG on PEEK substrate. Reproduced with permission from American Chemical Society under copyright CC BY 4.0 [26]

Polymers with aromatic structural compositions are widely used as carbonaceous substrates for the effective development of LIG. Upon laser exposure, the generated photothermal and photochemical phenomena rearranged the aromatic compound to develop a graphite structure [87]. The polymers, including polyimide (PI) containing phenyls, phenolic resins, and polysulfone, can be integrated into graphene due to similar structural chemistry (Fig. 3). The LIG can be easily developed from phenolic resin and will solubilize in organic solvents like ethanol and acetone. Hence, the obtained solution of phenolic resin having the desired viscosity can be easily formulated for diverse applications such as coating and doping phenolic resin. Polysulfone and polyphenylsulfone films are sulfur-enriched polymers, which can be used to develop sulfur-doped LIG films after treatment with a CO_2_ laser. The integration of sulfur into LIG creates more electroactive sites for promising electrochemical sensing applications [26, 88, 89]. In addition, 3D graphene can be developed by LIG printing based on the principle of laminated object manufacturing. However, due to limited energy absorption, various carbonaceous substrates cannot be transformed into LIG films in ambient conditions after single-pulse laser exposure. For example, non-aromatic polymers with lower absorption capability for laser energy are relatively converted into LIG in a more laborious way as compared to aromatic polymers. Polyether ether ketone and resin polymers are promising 3D materials and carbonaceous substrates for LIG development. Hence, incorporating 3D printing into LIG development was an interesting approach for developing intelligent sensing devices [90, 91]. The combination of the two strategies prospers in the manufacturing of 3D-printed sensing devices with self-monitoring characteristics. The developed LIG thin film is an active substance for the sensor design and can effectively self-monitor the deformation in real time, including stretching, bending, rotation, and abrasion. These designed sensors exhibited higher sensitivity, excellent response and recovery time, and promising reliability [92, 93].

### Natural materials

In the current decade, green electronics have gained huge popularity and interest in research. Generally, green electronics development is based on natural materials and their desired derived materials. Among natural materials, wood is an abundant and favorable substrate and is highly recommended for developing LIG [94, 95]. A study was conducted by Ye et al. to design LIG by CO_2_ laser irradiation on pine wood in the control environment. The inert atmosphere was suitable to reduce the ablation of pine wood. Furthermore, the lignin in wood has a significant role in the high-quality production of LIG owing to its abundant carbon content and aromatic structural composition. Besides wood, there are some other natural materials reported in the development of LIG, including potato, coconut, cellulose, and cloth. These precursor substrates are highly suggested for LIG development by applying multiple lasing strategies [96, 97].

**Fig. 4:**
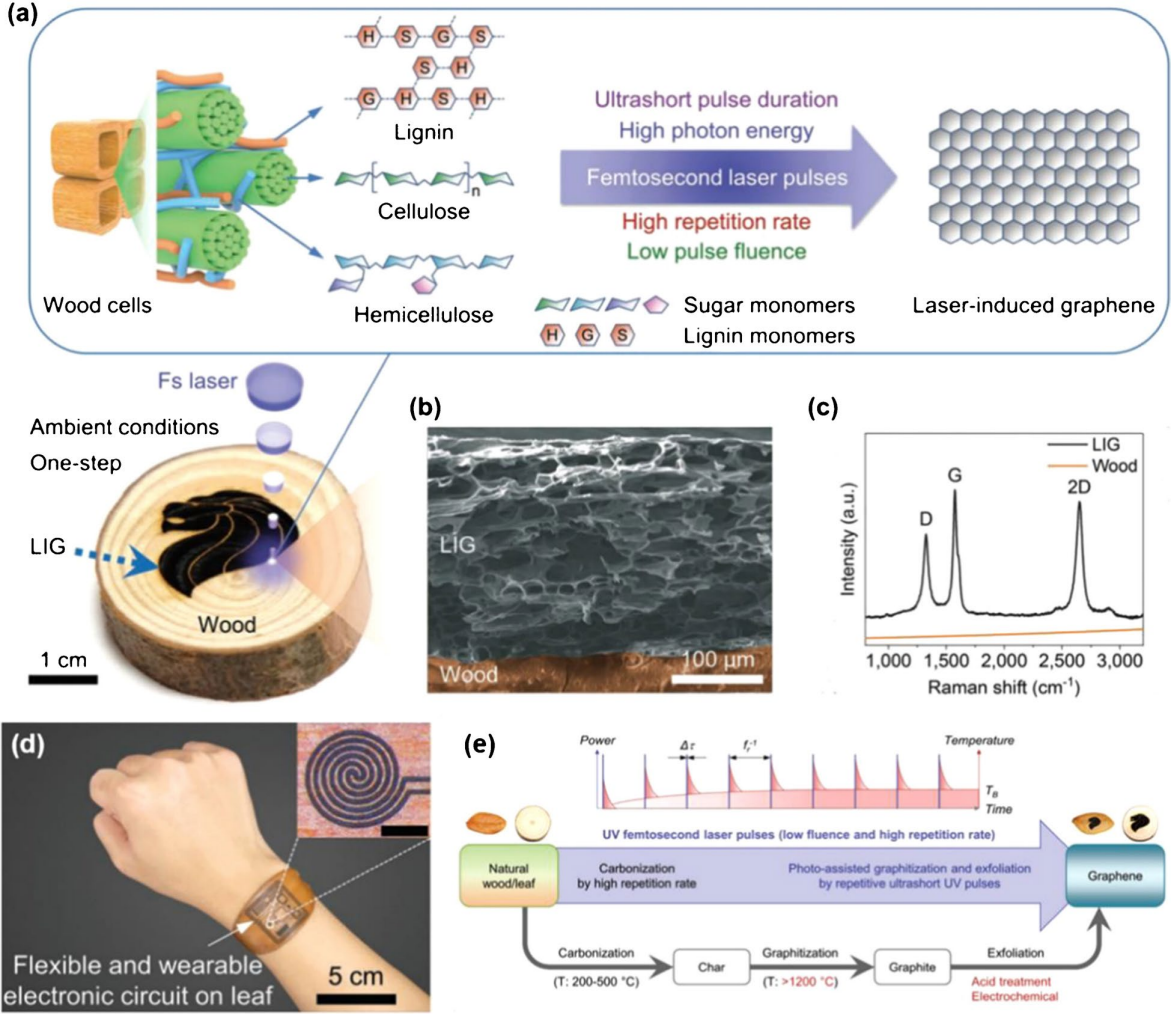
**a** LIG development on wood substrate. **b** SEM images of fabricated LIG. **c** Raman spectra of designed LIG. **d** Image of LIG printed electronic circuit on the leaf for developing flexible and wearable plant sensor devices. **e** Schematic illustration of LIG patterning mechanism. Reproduced with permission under copyright© 2019 WILEY‐VCH Verlag GmbH & Co. KGaA, Weinheim [100]

In another study, Le et al. developed LIG through the transformation of wood/leaf by UV laser exposure, which had high repetition and femtosecond pulsing speed. The femtosecond laser has a shorter pulse duration than thermal relaxation time, reducing thermal diffusion and minimizing substrate temperature. Furthermore, high repetition rate and laser energy restrict the heat into small areas by efficient light absorption. Hence, the femtosecond laser converts wood and leaves effectively into LIG by minimizing the ablation. In addition, femtosecond lasers are promising in developing LIG with high-resolution patterning. The transformation of wood and leaves into LIG is composed of two successive steps. Firstly, wood was carbonated into coke as an intermediate substrate by a thermal process generated by laser pulse repetition. Secondly, the intermediate substrate coke was transformed into LIG by graphitization along with an exfoliation process under the exposure of a very short laser pulse duration (Fig. 4) [98, 99].

## Sensing mechanisms and operational modes of LIG biosensors

**Fig. 5:**
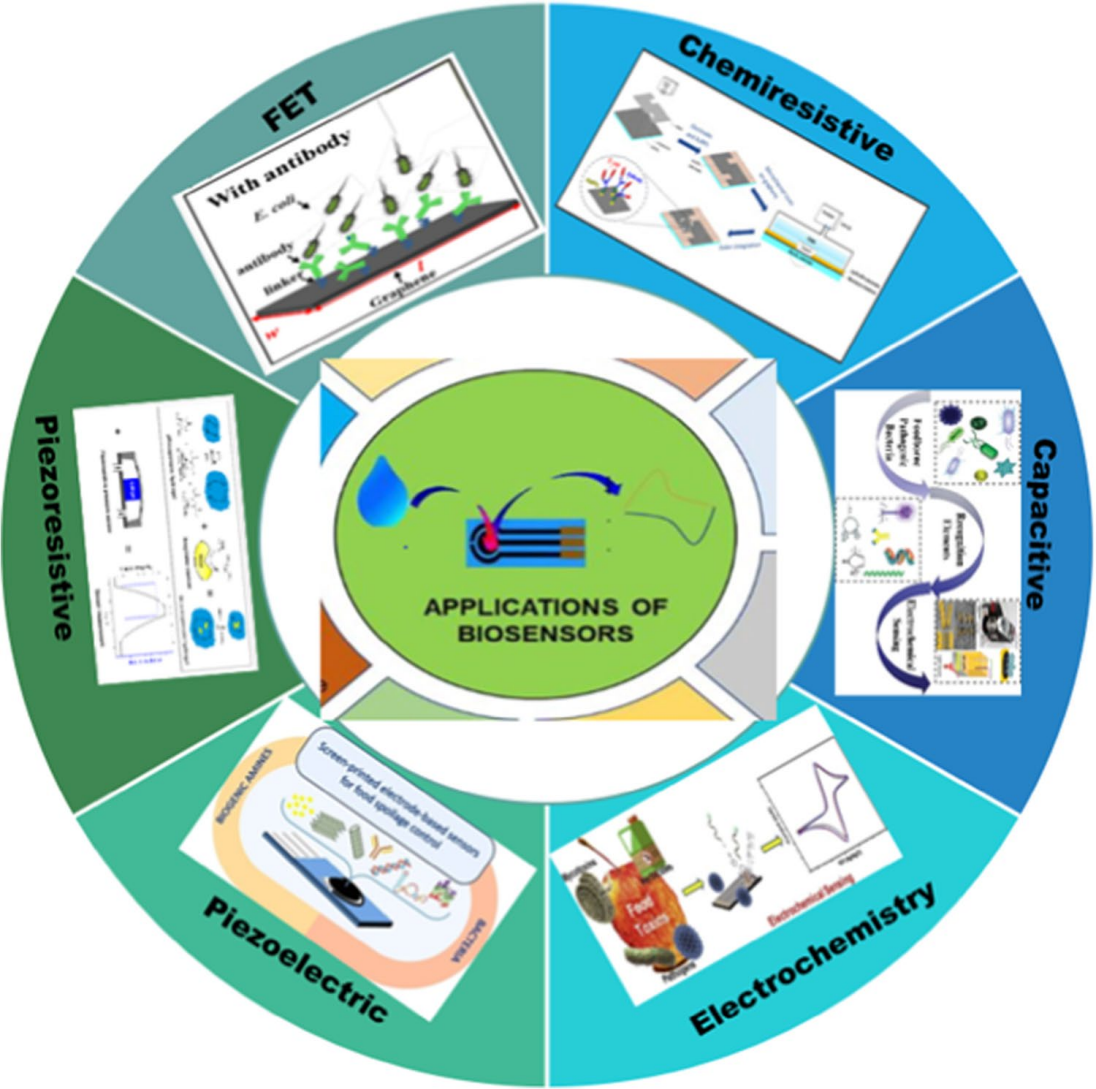
Sensing mechanisms of LIG biosensors

LIG-based sensors have different sensing mechanisms comprising capacitance, piezoresistive, piezoelectric, field-effect transistors, electrochemical, and chemiresistors to enable sensitive and selective detection of analytes. (Fig. 5). These sensing mechanisms explain the basic sensing principles of LIG-based different sensors [26, 101]. Electrochemical and resistive-based LIG sensors have high conductivity and porous nature to detect biomolecular interactions through changes in current, resistance, or capacitance. These sensing mechanisms are explained below exclusively. Electrochemical transduction is preferred due to fast response, miniature adaptability, and simple system, making it appropriate for onsite testing in food and dairy industries. But in case of complex food substrates, it may undergo interference. Capacitive and piezoresistive sensors present unlabeled detection with excellent mechanical response but are more likely to experience signal variations and environmental fluctuations. Field-effect transistors are restricted to signal amplification and real-time detection but are also restricted to microfabrication and surface optimization. While the simple and fast chemiresistive mechanisms also have limited selectivity, unable to differentiate isostructural food samples. That’s why, the chemical and physical nature of food samples, matrix intricacy, and optimal trade-off between reliability and sensitivity must be considered for the selection of sensing mechanisms [26, 102].

### Capacitive

A capacitive-LIG sensor is made of two conductive electrodes with a dielectric layer as an intermediate. Electrodes and dielectric layers predict the overall capacitance of developed sensors. Different complex structures are suggested for the dielectric layers, including pyramids, porous structures, and micropillar arrays, along with rough interfaces that have a key role in enhancing the sensitivity of sensors. However, the development of dielectric layer structures requires complicated processes. Hence, there is a great demand to develop methods that would be simple and cost-effective. Controlling the growth of microstructures is highly recommended to improve the sensitivity and response time of designed sensors. Furthermore, regulating the roughness of LIG electrodes can be used to enhance the sensitivity of capacitive-based biosensors [103–106].

### Piezoelectric

A piezoelectric effect based-LIG sensor works on the variation of dipoles among piezoelectric substances like polyvinylidene fluoride and zinc oxide under applied pressure, resulting in accumulating charge and formulating voltage on the surface of electrodes. Piezoelectric mechanism-based LIG sensors can transform mechanical energy into voltage signals, which facilitates the development of self-regulated sensor devices that are effective in wearable electronics and drug delivery systems. However, there are certain limitations, such as piezoelectric sensors having the capability to detect just dynamic pressure; hence, the piezoelectric substances only generate transient signals against external forces. The produced transient signal disappears soon, even in the presence of external forces [107, 108].

### Piezoresistive

To overcome abovementioned limitations in piezoelectric-based sensors, piezoresistive sensors are suggested, which transform external stimuli into resistance variations. Piezoresistive-based LIG sensors are widely applied because of their simple structure, easy readout features, broad-spectrum detection range, and excellent durability. The sensing mechanism of LIG-based piezoresistive sensors is mainly based on the effect of sublattice symmetry breach under the influence of uniaxial strain leading toward bandgap opening of LIG and enhancing the resistance. Moreover, conductive materials fragment networks integrated by graphene and its derived products can interact with each other under exposure to applied strain and pressure. The assembled LIG network in the absence of external force indicates reversible variations in resistance. In piezoresistive sensors, the sensing mechanism works by recording variations in contact resistance generated by cross-sectional distortion when exposed to external pressure. Hence, developing a microstructural array of LIG materials is of key importance for improving the resistance of designed sensors. In addition, the synthesis of porous graphene structures is equally important to enhance the sensitivity and rheological features of LIG sensors based on piezoresistive mechanisms [109–112].

### Electrochemical

In the previous decades, graphene was extensively used in biosensors due to its fast and heterogeneous transfer of electrons, broad-spectrum potential range, minimum overpotential, and low residual current. These attributes made graphene an interesting and promising candidate in electrochemistry. To enhance the electrochemical sensing abilities of graphene, the surface can be functionalized by covalent and non-covalent binding of functional groups, including hydroxyl, carboxyl, etc. The covalent modification is suitable for designed graphene with particular strength and excellent stability. However, this process is time-consuming and sensitive to synthetic environments. In comparison, non-covalent modification can be obtained by intramolecular interactions like hydrophobic and hydrogen bonding. Graphene is considered an aromatic molecule, and substances with aromatic rings can be integrated into the surface of graphene. Furthermore, the combination of different functional groups is a commonly applied strategy to obtain high-performance LIG sensors [113–116].

### Chemiresistive

Various LIG sensors are based on chemiresistive sensing mechanisms for detecting biological and gas molecules. In this mechanism, charge transfer occurs among graphene and absorbed biological molecules that alter the carrier density and resistivity of graphene. Graphene has greater carrier density, mobility, and minimum intrinsic noise features, making it promising for sensing different biological and gas molecules. The detecting molecules can be considered dopants, and their tunable properties can regulate the resistance of graphene. The specificity and sensitivity of graphene can be optimized for effective sensing. The modification of graphene enhances the adsorption, sensitivity, and selectivity of detecting molecules [117–120].

### Field-effect transistor

Field-effect transistors (FETs) are extensively applied in different sensors, including biosensors, gas sensors, and chemical sensors. FETs-based sensors are promising due to their ability to amplify the detection signal, easy and simple configuration, cost-effective, and real-time sensing. FET-based sensors have four components: gate electrode, source and drain electrode, conducting active layer, and dielectric layer. Graphene can be applied as an electrode and active layer in FETs-based sensors. However, the zero bandgap of graphene seriously limits its usage in biosensors. To attain a tunable bandgap of graphene, convert it into nanometer scale such as graphene nanomaterials or quantum dots. In addition, chemical modification, like functional groups on reduced graphene, also opens up the bandgap of graphene [121–125].

## LIG-based biosensors modifications

The modification of LIG-based biosensors is a key parameter for their applications in food and dairy products. Tunable attributes of LIG fabrication with controlled morphology, surface area, rheological, electrochemical, and physical can be generated under different laser irradiations. Doping with heteroatoms during the fabrication process enhances the performance of LIG-based biosensors to a greater extent. Similarly, modification of LIG with appropriate materials results in hybrids and composites having promising functions. Furthermore, the tunable spatial resolution makes them applicable for various applications. Multiple approaches have been suggested to attain the desired attributes of LIG-based biosensors by altering the laser parameters or adjusting the reduction conditions [126–128].

There are numerous approaches for LIG surface modifications, like doping of nanoparticles, antibody-based surface coating, and molecularly imprinted polymers. These methods enhance the selectivity and sensitivity of sensors, along with both pros and cons. Doping of nanoparticles like AuNPs and CuNPs also considerably raised the conductivity and electrocatalytic activity, but accumulation and instability have been observed due to recurring cycles. On the other hand, MIPs strategies, although lacking resilience in multiphase food matrices, are exceptional in selectivity because of molecular recognition strategies. Laser-induced graphene (LIG) functionalized with antibodies offers high specificity; however, its performance is limited by the susceptibility of antibodies to denaturation and reduced storage stability. To overcome these limitations, hybrid approaches—combining two or more sensing strategies with careful optimization—have shown promising potential for enhancing real-world applicability [5, 43].

The surface topography significantly impacts the surface area with active binding sites, surface attributes, and electrochemical activity of LIG-based biosensors. LIG surface morphologies can be optimized by adjusting the laser fluence and stacking density of the laser pulse. A particular fluence is suggested to enhance the carbonization process on the surface of the precursor substrate. Similarly, pulse stacking density is also a key element to attaining desired LIG surface morphology and structures. It is observed that greater stacking density of laser pulse generates sheet-like morphology, while lower stacking density of laser pulse produces fiber-type structure. Furthermore, continuous wave laser mode can be applied to create porous LIG structure with high strength, freestanding mode, and excellent electrical properties that make them effective in the development of biosensors [53, 126, 129].

The optimizations of surface attributes of LIG, particularly hydrophobic and hydrophilic properties, are key parameters and crucial for long-term applications of LIG-based sensors in food and dairy products. Conventional methods require different steps to design graphene materials with particular surface attributes and require chemical modification or surface coating in post-graphene modifications. LIG mostly showed hydrophilicity, and it can be optimized by carrying fabrication in gaseous atmospheres such as O_2_, air, argon, and helium. The LIG fabricated in different gaseous environments has different contact angles and affects the chemical properties of materials. Furthermore, the porous LIG structures also lead toward the super hydrophobicity of designed materials. The LIG microstructures with superhydrophobic properties are highly promising for electrochemical sensing due to enhanced interaction with analyte molecules, more binding sites, and increased defects to promote the transfer of free electrons [55, 130–132].

The application of LIG biosensors is based on particular electrical conductivity demands. The electrical conductivity of LIG is tunable and can be optimized by controlling the fabrication attributes, including laser power, exposure time, and writing speed. It is observed that electrical conductivity is excellent without limitation of dimensions; hence, sheet resistance has promising measurements due to the film’s limited thickness. The sheet resistance of the LIG film was reduced by increasing the laser power at ambient conditions. Similarly, laser scanning speed has a greater impact on the sheet resistance of LIG film. The low scanning speed with prolonged exposure time increases the temperature of the substrate and generates microcracks in LIG film, resulting in lower electrical conductivity. On the other hand, high scanning speed produced the best sheet resistance due to the weak photothermal effect. Furthermore, the tunable parameters to control conductivity are not only based on laser power, exposure time, and scanning speed, but any attributes that impact the heat accumulation in the development of LIG can be considered to optimize for the desired sheet resistance of LIG film [92, 133, 134].

The long-term stability, precision, and selectivity of LIG biosensors are vital for their promising and real-world applications in the food and dairy industry. Stability of LIG electrodes is particularly affected by environmental exposure like temperature, humidity, and electrochemical cycling. The laser parameter, substrate materials, and suitable doping elements can improve the structural robustness of LIG electrodes. Similar to stability, selectivity is one of the major challenges in complex food matrices sensing, resulting in non-specific binding. To cope with these challenges, surface functionalization with antibodies, aptamers, and molecularly imprinted polymers is suggested to ensure selective binding with particular analytes. Development of LIG electrodes with durable performance for longer periods and specific binding in the presence of structural analogues remains an active research area [135].

The modification approaches of LIG-based biosensors are vital for enhancing electroanalytical performances, encompassing sensitivity, selectivity, durability, stability, and response time. Hence, surface modifications and functionalization with heteroatom doping elements, composite development, and optimization of laser attributes enable the real-world applications of LIG biosensors. Like, heteroatoms doping particularly enhances the electron transfer kinetics and increases binding sites, improving the electrocatalytic activity. On the other hand, metallic nanocomposite integration into LIG promotes conductivity. It makes it suitable for selective and sensitive monitoring of analytes, improving the sensor’s detection limit. Furthermore, the tuning of laser parameters such as scanning speed, laser wavelength, and exposure time directly influences the porosity, surface area, and surface morphology of LIG electrode and has a greater impact on the electroanalytical performance of sensors [136].

## LIG biosensors applications in food and dairy industry

Graphene-derived biosensors have been studied for their application in the food and dairy industry. The increasing demand for sensing electronic devices has prompted the utilization of graphene and its derivatives as detection platforms for various food and dairy ingredients, toxins, microbial contaminants, and biological fluids [137]. Graphene has unique rheological and physicochemical properties and has emerged as a promising material in biosensor devices, particularly in food and dairy-associated applications. The graphene-based sensors have excellent sensitivity, selectivity, and precision in detecting traces of food ingredients and adulterants or contaminants [138, 139]. The modification of graphene to improve the adsorption of biomolecules is an excellent strategy for the sensitive detection of food analytes. The graphene modified with carboxyl functional groups generates a platform for immobilizing food molecules during sensing [92, 140]. In addition, the reduced graphene oxide modified with functional groups is excellent for attaching food molecules with promising conductivity. It proves its application in the sensing of food and dairy products [141, 142].

LIG-based biosensors have been utilized for various food and dairy industry applications. Various sensing strategies have distinct advantages and applications against different food and dairy industry analytes [143]. Electrochemical-based LIG sensors are most commonly used in this field due to ease of fabrication, simple miniaturization, good sensitivity and selectivity, and lower limit of detection for antibiotics, biogenic amines, and food contaminants [144, 145]. On the other hand, FET-based LIG sensors are effective for label free detection and high signal amplifications of analytes molecules. Meanwhile, chemiresistive sensors offers simple and fast response [146].

LIG sensors exhibit significant potential for food and dairy analysis, as they offer significant detection of a wide range of analytes such as pathogens, biogenic amines, additives, and antibiotic remains. Electrochemical platforms are highly preferred for their excellent sensitivity, simple and easy integration, but they show a decline in consistent performance in complex food matrices due to matrix interference and non-target interaction in real samples. While colorimetric and photoelectrochemical approaches usually provide simple visualization or signal enhancement, they mostly show a lack of sensitivity and limitations that are compulsory for the detection of trace contaminants. Although molecularly imprinted polymers and LIG sensors coated with antibodies reveal significant specificity and selectivity towards targeted samples, their life span, reliability, and reproducibility show limitations in real-world environments and field work. The emergence of wireless and wearable biosensors reflects a promising trend towards smart packaging and real-time detection of decaying food. Still, there are issues related to productivity at a larger scale; long-term stability is a concerning point and requires more enhancement, despite their versatility, adaptability, and promising potential for monitoring the safety of food. The successful commercialization of LIG-based biosensors needs more meticulous optimization and refinement of design, improving their performance [147].

A detailed overview of different graphene sensors in the food and dairy industry has been described briefly in Table 2, presenting designs of LIG-based sensors specific to nutrients, antibiotics, pathogens, and pollutants in dairy products and food. Biosensors have the capability of quick detection with low limits and specificity enhancement due to conjugation with antibodies or nanoparticles. This table also highlights the adaptability and high performance of LIG sensors for food analysis in the real world.

**Table 2:** List of LIG-based biosensors for detecting various food and dairy analytes

Sensor configuration	Analyte	Sensing techniques	Linear concentration range	Limit of detection (LOD)	Response time	Fabrication	Reference
Ru(bpy)_3_^2+^	*Cryptosporidium parvum*	Electrochemiluminescent	-	3 pmol L^−1^	15 min	Laser scribing on PI modified with Ru(bpy)3^2^⁺-liposome complex and DNA	[147]
LIG electrode functionalized with antibodies	*Salmonella enterica*	Electrochemical Immunosensor	25 to 105 CFU mL^–1^	13 ± 7 CFU mL^–1^	22 min	LIG fabricated on PI functionalized with antibodies	[8]
LIG integrated immunoassay strip	*Salmonella typhimurium*	Colorimetric	1 CFU/10 mL– 10^8^ CFU/10 mL	1 ± 0.5 CFU/10 mL	12 min	Laser scribing on PI and integration with gold nanoparticles	[73]
Cu/LIG	Glucose	Electrochemical	0.005–0.525 mM	5 μM	2 s	Laser-induced patterning and modification with Cu_2_^+^/ethyl-cellulose	[76]
LIG chip	Naringin Hesperidin	Electrochemical	50 nM–100 μM	11 nM 15 nM	-	Laser scribing on PI and used for CV measurements	[148]
LIG/Ag electrode	Formaldehyde	Electrochemical	0.05–5 μg/mL	0.011 μg/mL	3 min	Laser engraving on PI and electrodeposition of AgNPs	[149]
(AuNPs)-polypyrrole-chitosan-LIG	Vitamin C	Electrochemical	0.25–5.00 mmol L^−1^	0.22 mmol L^−1^	2 min	Laser ablation on PI and hybridization with AuNPs	[78]
NanoPd@LIG	Formaldehyde	Electrochemical	0.01–4.00 mM	6.4 μM	-	PI integration with Pd_2_^+^ and chitosan followed by laser induction	[79]
Cu-LIG/ITO	Vanillin	Electrochemical	0.25–40 μg/mL	0.14 μg/mL	4 min	LIG integrated with Cu and deposited on ITO electrode via laser ablation	[81]
Cu-plated LSG	Biogenic amines	Electrochemical	19.24 ± 8.21 mg histamine/kg	11.6 µM	7.3 s	Laser scribing on PI and deposition of Cu functionalized with diamine oxidase	[150]
LIG ion-selective electrode	Nitrite	Electrochemical	10^−5^–10^−1^ M	0.3 ± 0.1 mg L^−1^	3 min	Laser engraving on PI and modified with PVC based nitrite	[151]
LIG-based MIP sensor	Tetracycline	Electrochemical	10–300 nM	0.85 nM	-	Laser scribing on PI and MIP with phenylenediamine monomer and tetracycline template	[2]
AuNSs/LIPG	Sulfonamide	Electrochemical	0.4–100 μM	0.035 μM	-	Laser direct writing on PI, decorated with gold nanoshells	[152]
AuNPs/LIG electrode	Tigecycline	Electrochemical	0.01 to 800 nM	0.003 nM	3 min	Laser engraving on PI functionalized with AuNPs and MIP layer of phenylenediamine	[153]
h-BN/LIPG	Sulfonamides	Electrochemical	0.5–362.5 µM	0.011 µM	4 min	LIG electrode engraving deposited with 2D h-BN nanosheets	[154]
LIG/PbS/CdS	Ampicillin	Photoelectrochemical (PEC)	5.0–2 × 10^4^ pM	1.36 pM	6 min	LIG electrode decorated with PbS/CdS and CoOOH nanosheets, via laser	[155]
Ag-La(OH)_3_@Dy_2_O_3_	Bisphenol A Tartrazine	Electrochemical	-	9.2 nM 0.96 nM	-	Ag-La(OH)_3_@Dy_2_O_3_ nanocomposite integrated with LIG through laser ablation on PI	[156]
Au/LIG electrode	Organophosphorus	Electrochemical	3–400 ng/mL	1.2 ng/mL	3 min	LIG electrode deposited with AuNPs via electrodeposition	[157]
Pt/LIG	Glyphosate	Electrochemical	10–260 µm	0.991 nA µm^−1^	4 min	Laser engraving on PI and deposition of Pt nanoparticles	[158]

### Pathogens detection

Food-borne diseases are a growing concern among food and dairy product consumers, with increasing numbers of cases every year. Hence, developing fast, cost-effective, simple, and sensitive approaches for on-site pathogen detection to combat problems is highly recommended. Laser-induced graphene sensors have great potential for pathogens at low limits of detection in food and dairy products [159]. LIG electrodes are integrated into microfluidic channels to investigate different detection approaches, including electrochemical and electro chemiluminescent. These strategies are simple, cost-effective, and mandatory for the development of assays in point-of-care sensing devices [160, 161].

A study was conducted by Gerstl et al. [147] to develop an LIG sensor based on electrochemical and electro chemiluminescent sensing mechanisms for the detection of pathogens. He investigated electrode design, dimension of the microfluidic channel and assay based on DNA hybridization along with liposomes for signal enhancement. The developed sensor was used to detect the DNA of the pathogen *Cryptosporidium parvum*. Liposomes entangled Ru(bpy)_3_^2+^ to generate electro chemiluminescent signal or K_4_[Fe(CN)_6_] for electrochemical detection. The designed microchip has promising analytical features for sensing applications. It has a low detection limit of about 3 pmol L^−1^ for electro chemiluminescent sensing and 47 pmol L^−1^ for electrochemical sensing. The developed sensor was highly specific toward the targeted DNA analyte. This strategy was successfully applied for the on-site detection of pathogens [147].

**Fig. 6:**
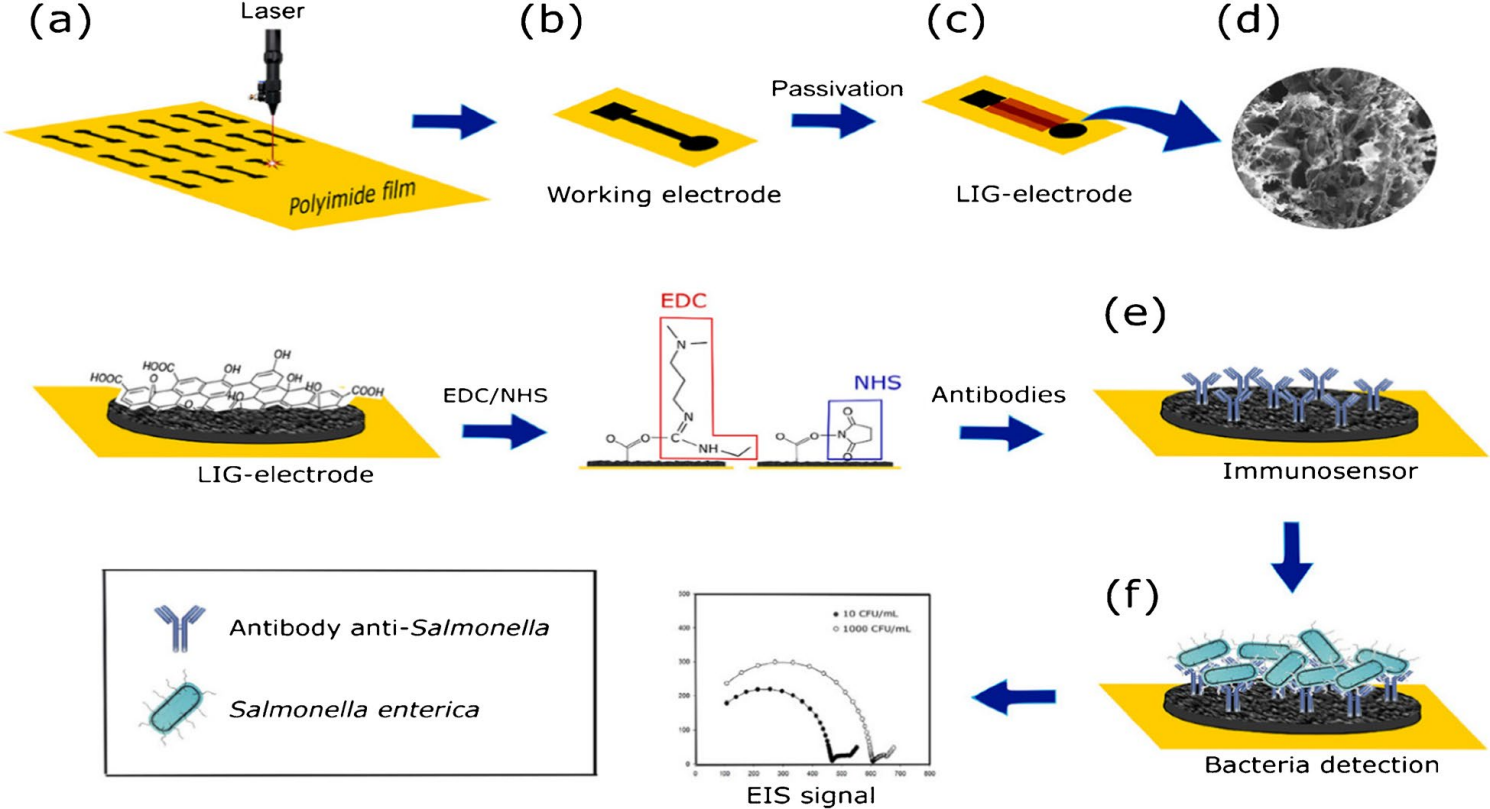
Fabrication, functionalization, and sensing mechanism of LIG biosensor. **a** Development of LIG on polyimide sheet (PI). **b** LIG working electrode. **c** Passivation of LIG electrode with lacquer. **d** SEM image of fabricated LIG electrode. **e** Immobilization of Salmonella antibody on the surface of LIG electrode. **f** Attachment of salmonella to the modified LIG electrode and electrochemical sensing Nyquist plot. Reproduced with permission from American Chemical Society under copyright CC BY 4.0 [8]

In another study, Soares et al. [8] designed LIG electrochemical immunosensors to detect the pathogen *Salmonella enterica* in food samples. He fabricated a label-free and sensitive LIG electrode functionalized with particular antibodies to detect the food pathogen *Salmonella enterica* electrochemically. The LIG electrodes were developed through laser induction in an ambient environment. After LIG functionalization with specific Salmonella antibodies, the designed sensors sense the live Salmonella in chicken broth. This sensor has a low limit of detection of about 13 ± 7 CFU mL^–1^, along with a wide linear range of 25 to 105 CFU mL^–1^. It had a response time of 22 min and required no sample preconcentration. Furthermore, the developed LIG immunosensor exhibited higher selectivity by showing no significant response to related bacterial strains (Fig. 6). Hence, the developed LIG sensor can be used effectively for rapid, label-free, and cost-effective detection of pathogens in food and dairy products [8].

**Fig. 7:**
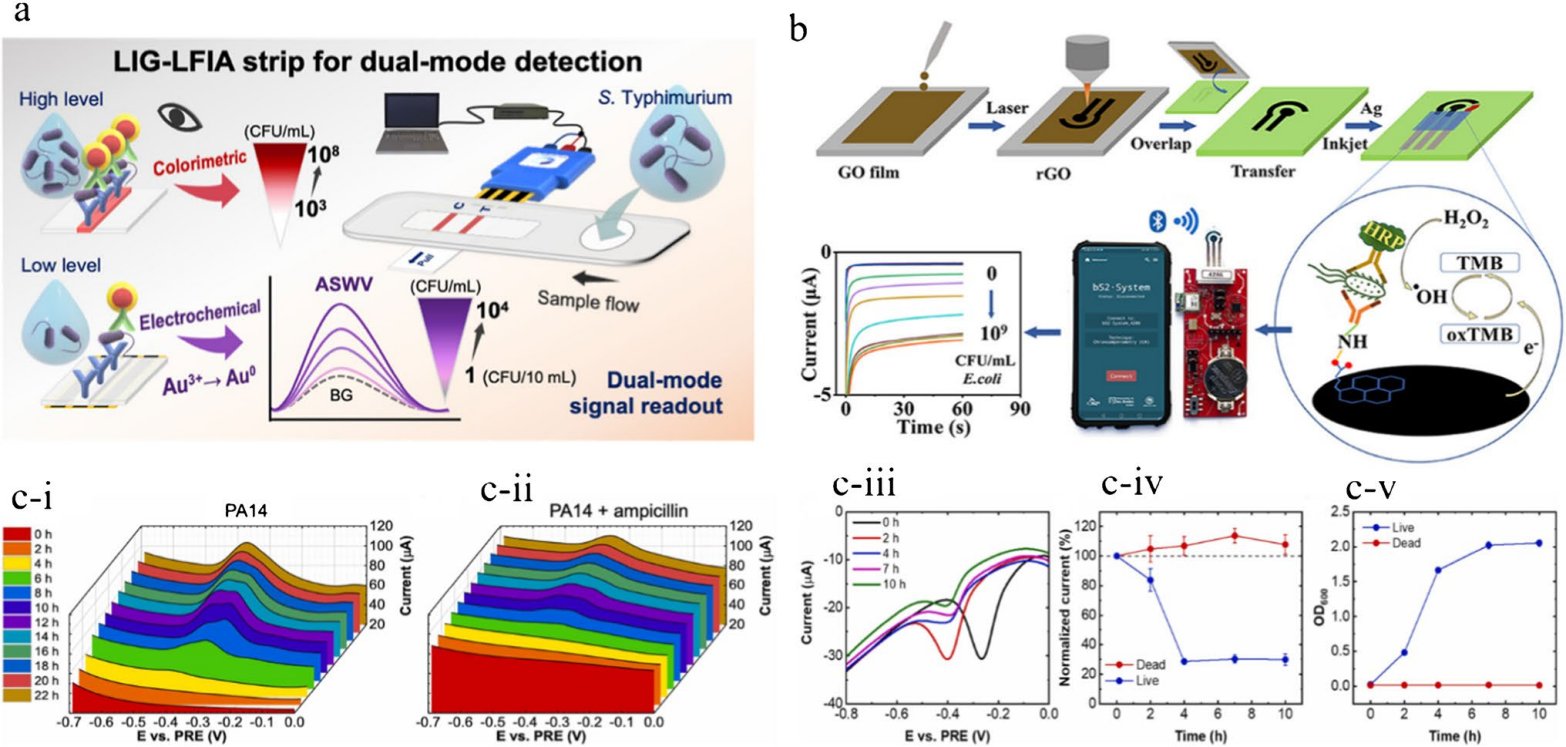
**a** Pictorial representation of Salmonella sensing by colorimetric and electrochemical method via LIG integrated immunoassay strip. Reproduced with permission from American Chemical Society under copyright CC BY 4.0 [73]. **b** Graphical explanation of *E. coli* sensing by laser-reduced graphene oxide modified electrode. Reproduced with permission from American Chemical Society under copyright CC BY 4.0 [74]. **c** Detection of *Pseudomonas aeruginosa* culture on LIG. c-i) SWV graph of PA14. c-ii) PA14_amp_ represents the peak after 22 h of testing. c-iii) Metabolic activity assay of *E. coli* on resazurin. SWV graph for live *E.coli* culture. c-iv) Normalized current graph for *E.coli* live and heat-killed. Error bars indicate the standard deviation. c-v) The OD600 value of *E.coli* culture having no resazurin, and error bars show standard deviation. Reproduced from Elsevier under copyright © 2023 Elsevier B.V. [75]

Preechakasedkit et al. [73] developed a colorimetric and electrochemical methods based on an LIG integrated immunoassay strip for the effective sensing of *Salmonella typhimurium*. In this study, the LIG electrode was produced by laser induction on polyimide film with a pseudo silver/AgCl reference electrode from silver sintering and chlorination. Automatic reagent supply was achieved for dual mode reading with single sample loading. For colorimetric signals, the gold nanostructures deposited approach was used to enable early screening and electrochemical response for higher sensitivity and quantification. The designed biosensor chip has an excellent performance, indicated by rapid analysis (12-min assay), wide spectrum working concentrations in the range of 1 CFU/10 mL–10^8^ CFU/10 mL, and a lower limit of detection of about 1 ± 0.5 CFU/10 mL (Fig. 7a). This sensor strip was successfully used in the detection of different food pathogens without bacterial amplification, and the obtained results were in good agreement with standard culture test [73].

Zhao et al. [74] developed a laser-reduced graphene oxide electrode for sensing food pathogen *E. coli.* In this study, he produced reduced graphene oxide-modified electrodes using a simple, cost-effective, and fast method by applying direct writing encompassing laser scribing and inkjet printing coupled with a stamp-transferring strategy. In this approach, graphene oxide was reduced and patterned when exposed to the laser, followed by press-stamping to a polyester sheet. The fabricated electrode was studied using different characteristic techniques, including SEM, XPS, Raman spectroscopy, and electrochemical sensing. The designed sensor was used for the electrochemical sensing of *E. coli.* This sensor has a wide concentration range of 917–2.1 × 10^7^ CFU/mL with 283 CFU/mL and a low limit of detection (LOD) (Fig. 7b). The sensor was coupled with a smartphone through a portable wireless system. Hence, the developed biosensor can be used for real-time detection of pathogenic food bacteria [74].

Another study was conducted by Butler et al. [75] to develop cellulose-derived LIG devices for monitoring bacterial contaminants by their metabolites. In this study, cellulose-derived LIG thin film with a resistance of 43.7 ± 2.3 Ω sq^−1^ was developed and used in electrochemical paper-based sensors. In the first step, time depended on the detection of pyocyanin excreted by *Pseudomonas aeruginosa* from planktonic culture and grown on an LIG paper-based chip was conducted. Remarkable variations were observed in the production of phenazine from bacterial cells cultured on LIG chips compared to planktonic culture. In the second step, *E. coli* cell viability and metabolic activity were studied by applying an electrochemical assay based on resazurin (Fig. 7c). This study demonstrated the application of cellulose-derived LIG sensors for the sensitive and effective monitoring of bacterial strains and provided new insight for the testing of food and dairy products’ pathogenic microbial species [75]. Kumari et al. designed a smartphone-based electrochemical sensor fabricated with a nanoflower for rapid, on-site, and sensitive quantification of hydrogen peroxide in milk samples. This was a novel approach used for the non-enzymatic sensing of hydrogen peroxide in food samples [162].

### Food ingredients

The sensitive sensing of functional ingredients in food and dairy products is important in the modern food and dairy industry. Rapid determination of food nutrients, including vitamins, flavonoids, protein, carbohydrates, formaldehyde, and mineral salts, is crucial for nutrition uptake and health care. The excessive consumption of any ingredients leads to severe health problems such as obesity, cancer, metabolic disorders, diabetes, and cardiovascular diseases[163, 164]. Hence, the precise monitoring of food ingredients is mandatory to prevent health issues and facilitate healthcare management. Recently, laser-induced graphene has garnered huge attention in food ingredients sensing owing to its physicochemical and rheological properties [126, 165, 166].

**Fig. 8:**
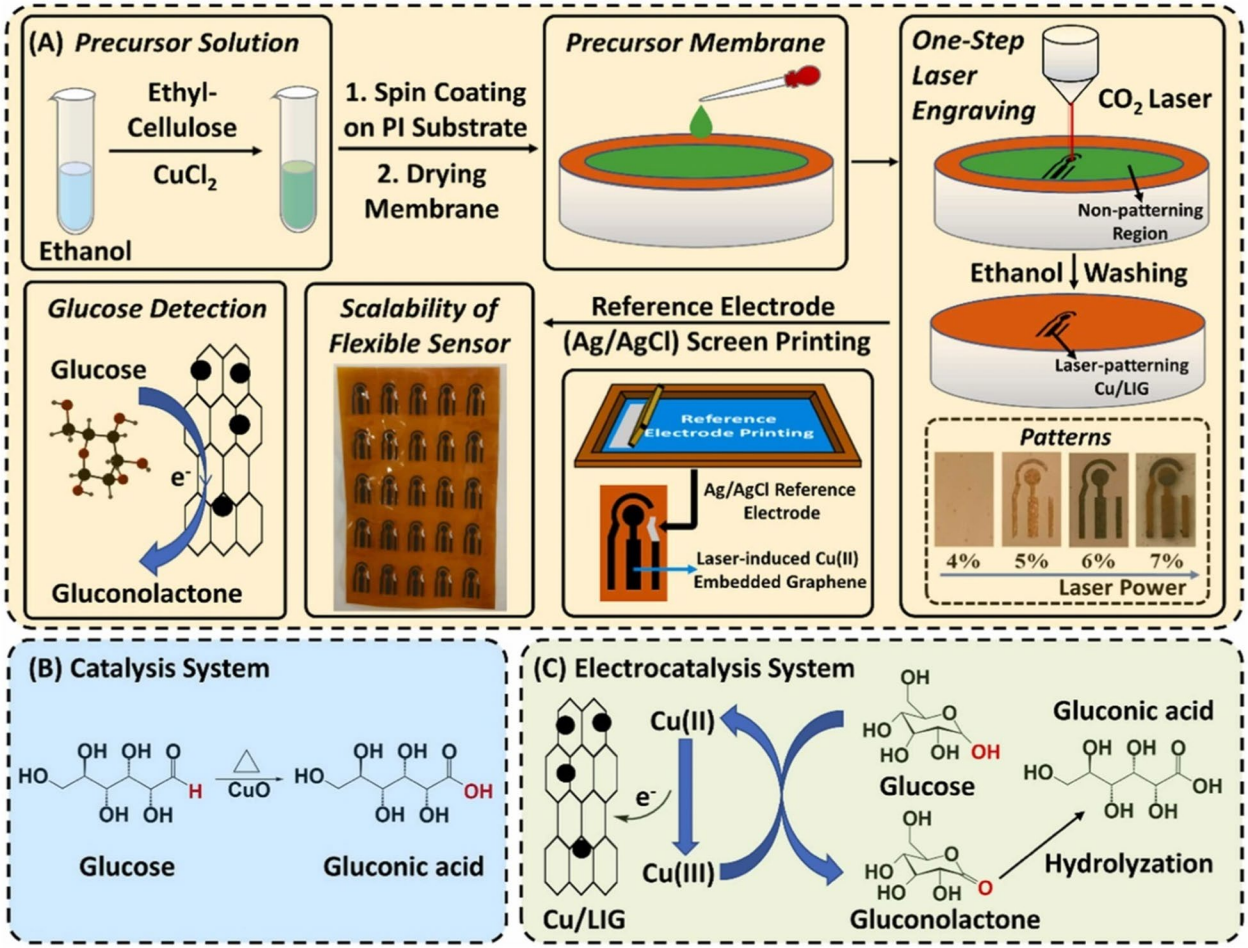
**A** Schematic illustration of Cu/LIG fabrication through spin coating followed by laser induction on the substrate membrane. **B** Glucose oxidation mechanism during catalysis under heating. **C** Glucose oxidation in the electrochemical sensing system. Reproduced from Elsevier under copyright © 2024 Elsevier B.V. [76]

Zhang et al. [76] designed a laser-induced copper-incorporated graphene sensor for non-enzymatic sensing of glucose molecules in beverages. During this study, an integrated LIG electrode was fabricated for rapid sensing of glucose molecules by direct laser patterning on Cu^2+^/ethyl-cellulose/polyimide sheet. The results indicated that the Cu/LIG fabricated electrode showed lower resistance for charge transfer and particular diffusion mechanisms. The improvement in resistance can be attributed to optimize LIG fabrication, resulting in the embedding of ethyl cellulose (EC) and copper chloride (CuCl_2_) as combustion substrates. The designed Cu/LIG electrode depicted promising electrochemical activity for glucose sensing with a limit of detection of about 5 μM, a response time of around 2.0 s, and a wide concentration range of 0.005–0.525 mM. In addition, the fabricated sensor showed higher selectivity, sensitivity, and stability (Fig. 8). This sensor was successfully applied to detect glucose in different beverages, including Cola, Sprite, Soda-Water, and Black Tea, manifesting their potential applications in the food and dairy industry [76].

**Fig. 9:**
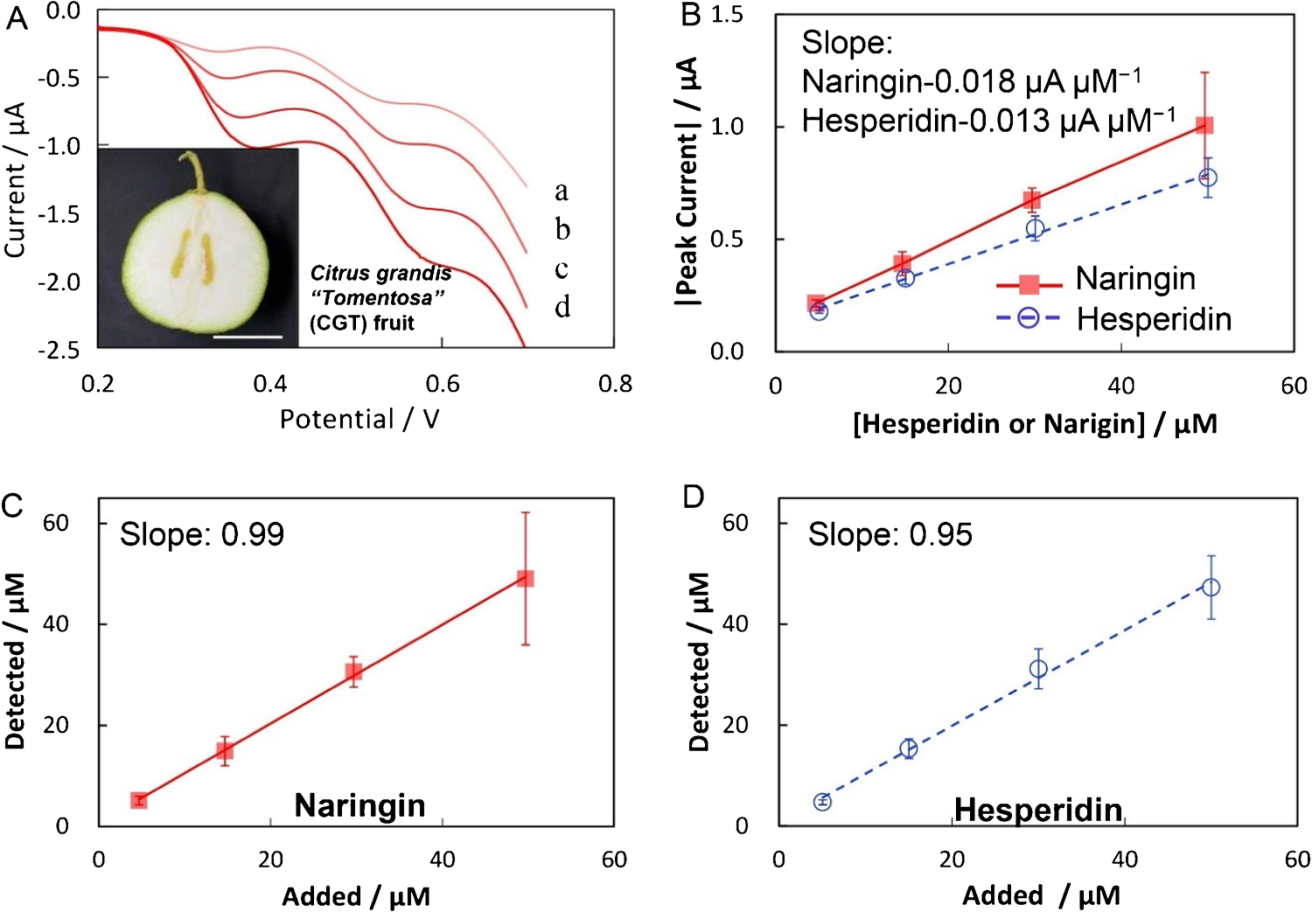
Sensing of naringin and hesperidin in citrus fruit. **A** Linear sweep voltammetry (LSV) of LIG-based electrochemical chip in PBS buffer at different concentrations of naringin and hesperidin. **B** Current values against the concentration of naringin and hesperidin. **C and D** Show the association between the monitored and added load of bioflavonoids. Reproduced from Elsevier under copyright © 2023 Elsevier B.V. [148]

Another study was conducted by Xia et al. [148] for the development of LIG chips for the effective sensing of bioflavonoids in fruits. In this study, a chip was designed by LIG at a polyimide sheet for the simultaneous and rapid sensing of bioflavonoids present in citrus fruits. During this study, the impacts of laser power on surface morphology and electrochemical properties of LIG were observed. In addition, at the LIG electrode, electrochemical sensing of naringin and hesperidin, along with kinetic analysis, was investigated to comprehend the detail sensing mechanism. Finally, a LIG chip was fabricated for rapid sensing of naringin and hesperidin. The designed LIG chip indicated a promising low limit of detection (11 nM for naringin and 15 nM for hesperidin), wide concentration range (50 nM–100 μM), higher selectivity, and good reproducibility. Moreover, the designed sensor chip was successfully applied for flavonoid detection in a real sample of citrus grandis with excellent reliability as well as validity (Fig. 9) [148].

**Fig. 10:**
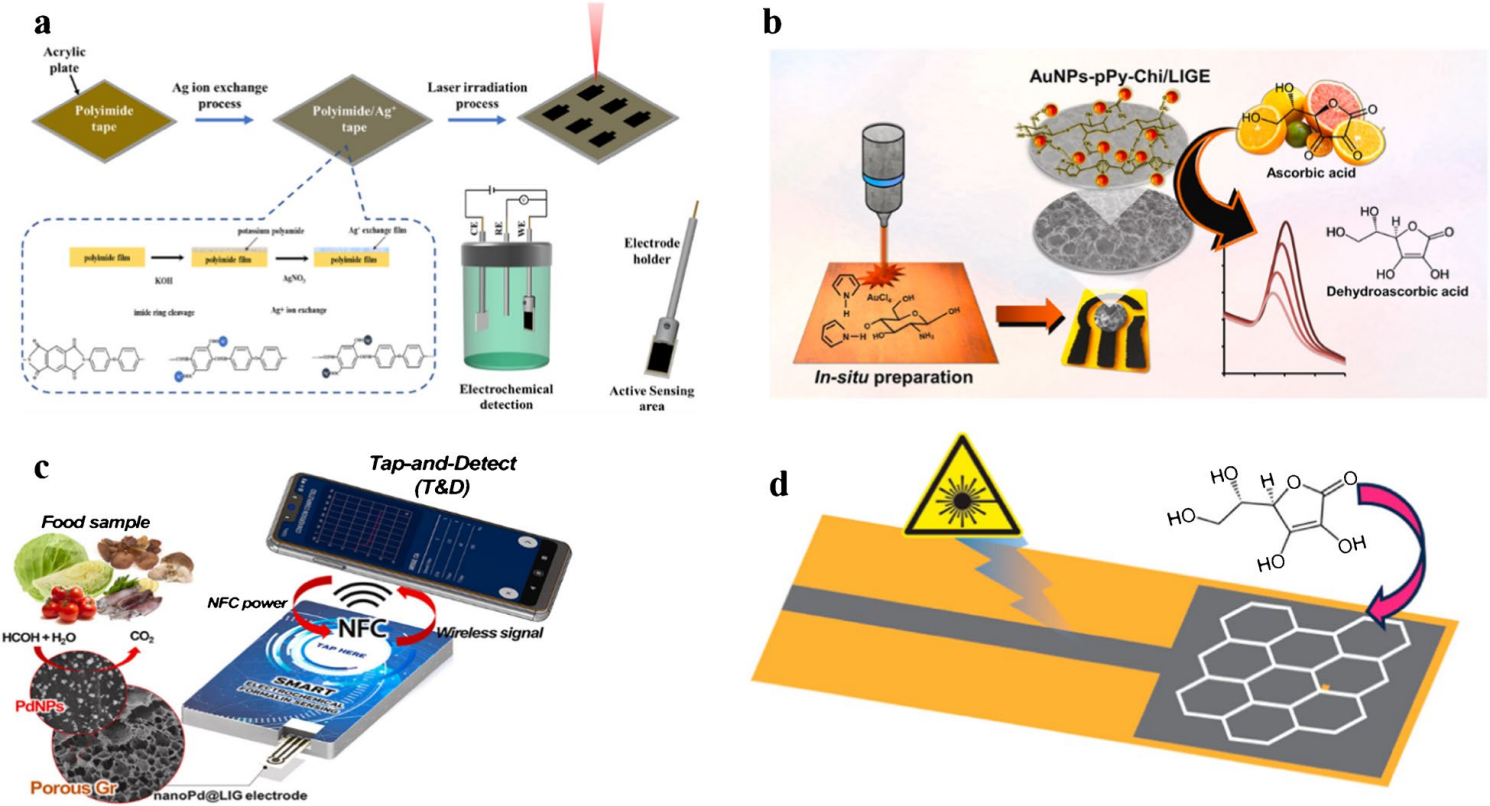
**a** Schematic demonstration of the development of LIG/Ag modified electrode for the sensing of formaldehyde. Reproduced with permission from American Chemical Society under copyright CC BY 4.0 [149]. **b** Graphical explanation of (AuNPs)-polypyrrole-chitosan-LIG-based sensor development for the sensing of vitamin C. Reproduced from Elsevier under copyright © 2024 Elsevier B.V. [78]. **c** A smart wireless sensor based on nanoPd@LIG modified electrode for the sensing of formaldehyde. Reproduced from Elsevier under copyright © 2022 Elsevier B.V. [79]. **d** Development of LIG electrode functionalized with porphyrin/ZnO for non-enzymatic sensing of ascorbic acid. Reproduced with permission from American Chemical Society under copyright CC BY 4.0 [80]

Chen et al. [149] designed a LIG-coated Ag nanoparticle electrode for the sensing of formaldehyde. In this study, a novel sensor made of an LIG electrode was fabricated using a laser induction method. The precursor substrate polyimide was optimized by a metal ion exchange approach, and the working electrode was deposited with graphene/Ag nanoparticles. The structural characteristics of the LIG-coated Ag electrode indicated a higher surface area, hence providing more binding sites for electrochemical sensing of formaldehyde. In addition, the differential pulse voltammetry (DPV) results indicated greater linearity against the varying formaldehyde concentration between 0.05 and 5 μg/mL and had a low limit of detection about 0.011 μg/mL (Fig. 10a). The designed electrode can be applied in a practical way by developing a sensor device for the real-time detection of formaldehyde in food and dairy products [149].

Similarly, another study was conducted by KongKaew et al. [78] to develop gold nanoparticles (AuNPs)-polypyrrole-chitosan on LIG for sensing vitamin C (ascorbic acid). During this study, the simultaneous fabrication of AuNPs and graphene was conducted on a polymer substrate via the laser ablation method. The Au precursor was synthesized in a solution of copolymer comprising pyrrole and chitosan, which improved the nucleation of Au on the LIG electrode. The structural and electrochemical attributes of the fabricated electrodes were investigated using SEM and cyclic voltammetry, respectively. The DPV measurements were performed at optimized parameters to improve the detection of ascorbic acid. The designed electrode exhibited two different linear ranges between 0.25–5.00 and 5.00 to 25.00 mmol L^−1^ and a lower limit of detection of 0.22 mmol L^−1^. This electrode was successfully applied for the detection of ascorbic acid (vitamin C) in orange juice with excellent reproducibility and recovery rate (Fig. 10b) [78].

Saleh et al. [79] developed a nano-palladium embedded LIG (nanoPd@LIG) electrode integrated with a wireless smartphone for the sensing of formaldehyde. The nanoPd@LIG composite was designed by single-step laser induction on a Pd^2+^-chitosan-polyimide sheet. The physicochemical properties of synthesized electrodes were investigated. In this study, the electrochemical sensing of formaldehyde at the nanoPd@LIG electrode was examined. The fabricated electrode was connected to a wireless mobile device for real-time monitoring of formaldehyde. This designed smart electrochemical sensing device is based on near-field communication mechanisms to receive power and transfer data to a smartphone. In addition, it showed a wide detection range between 0.01 and 4.00 mM and a lower detection limit of 6.4 μM with promising reproducibility and selectivity (Fig. 10c). The designed sensor was used to detect formaldehyde in actual food samples, and the results obtained were in agreement with potentiostat [79].

Another study was conducted by Pushparaj et al. [80] to develop an LIG electrode functionalized with porphyrin/ZnO for non-enzymatic sensing of ascorbic acid (vitamin C). In this study, the designed electrode was investigated as an extended gate field effect transistor (EGFETs). The sensitivity of LIG electrode modified with tetraphenylporphyrin/ZnO nanoparticles was higher than that of bare LIG. In addition, the limit of detection of the designed sensor was about 3 nM. This sensor also exhibited excellent selectivity by showing the signal response for ascorbic acid 250 times higher as compared to the same concentration of structural analog dopamine analyte. Furthermore, the developed sensor was used to sense ascorbic acid in real-food samples (Fig. 10d). Hence, this sensor can be applied effectively for commercial applications in the study of vitamin-rich food and dairy products [80].

**Fig. 11:**
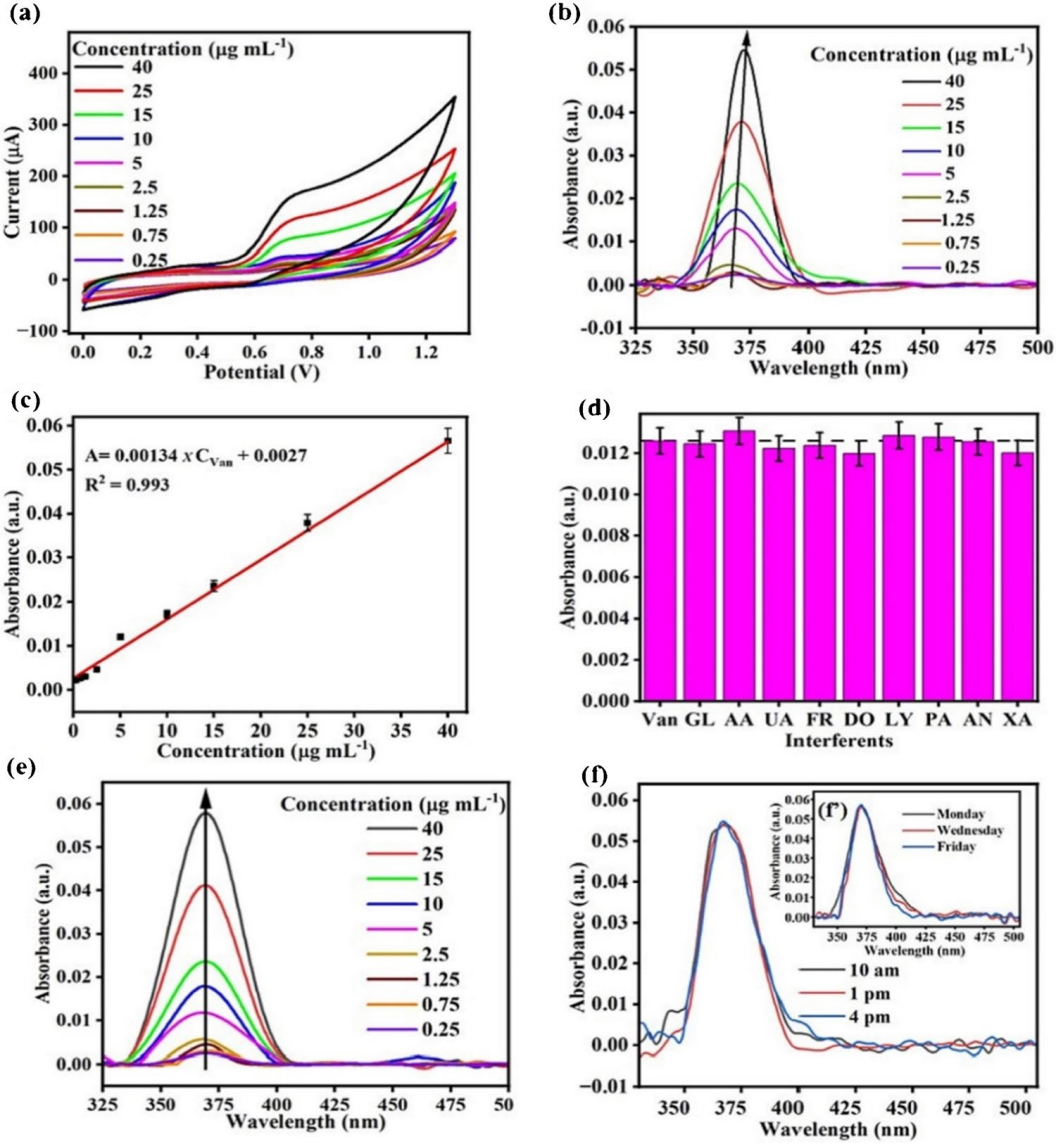
**a** Cyclic voltammetry (CV) response. **b** UV–visible spectra after 02 CV cycles. **c** Calibration curve for Cu-LIG/ITO modified electrode against different concentration of vanillin. **d** Absorbance values for vanillin in the presence of structural analog interfering molecules. **e** Selectivity of vanillin at different concentrations with interfering analytes. **f** Intraday and interday vanillin measurements. Reproduced from Elsevier under copyright © 2024 Elsevier B.V. [81]

A study was conducted by Gupta et al. [81] to develop a Cu-LIG nanocomposite for the sensing of vanillin in food samples. In this study, Cu-LIG nanocomposites were fabricated on indium tin oxide (ITO) as a working electrode. The designed material had higher surface area and conductivity, facilitating enhanced electrochemical sensing for the detection of vanillin. The fabricated Cu-LIG/ITO electrode was used to sense oxidized species of vanillin. The developed sensor had a broad linear concentration range from 0.25 to 40 μg/mL and a low limit of detection of 0.14 μg/mL (Fig. 11). Furthermore, this sensor exhibited promising selectivity for vanillin in the presence of structural analogue interfering analytes such as glucose, fructose, uric acid, and ascorbic acid. These results suggested that this strategy can be applied to the sensing of food and dairy ingredients in actual samples [81].

### Food spoilage

Observing the food texture, flavor and safe packaging are mandatory measures to prevent food spoilage and food poisoning. In addition, the real-time detection of temperature, humidity, and gas emissions from food and dairy products reduces the risk of spoilage because temperature, humidity, and gas can predict food status and precise shelf-life. Furthermore, food and dairy products decompose easily if not preserved and stored at suitable temperatures. Hence, it is especially important to monitor food status before intake. Smart, precise, and real-time monitoring of food and dairy products will not only prevent food poisoning but also improve storage and shelf-life [167, 168]. LIG sensors can effectively monitor the food and dairy product’s status with promising sensitivity, selectivity, and low limit of detection [169–171].

**Fig. 12:**
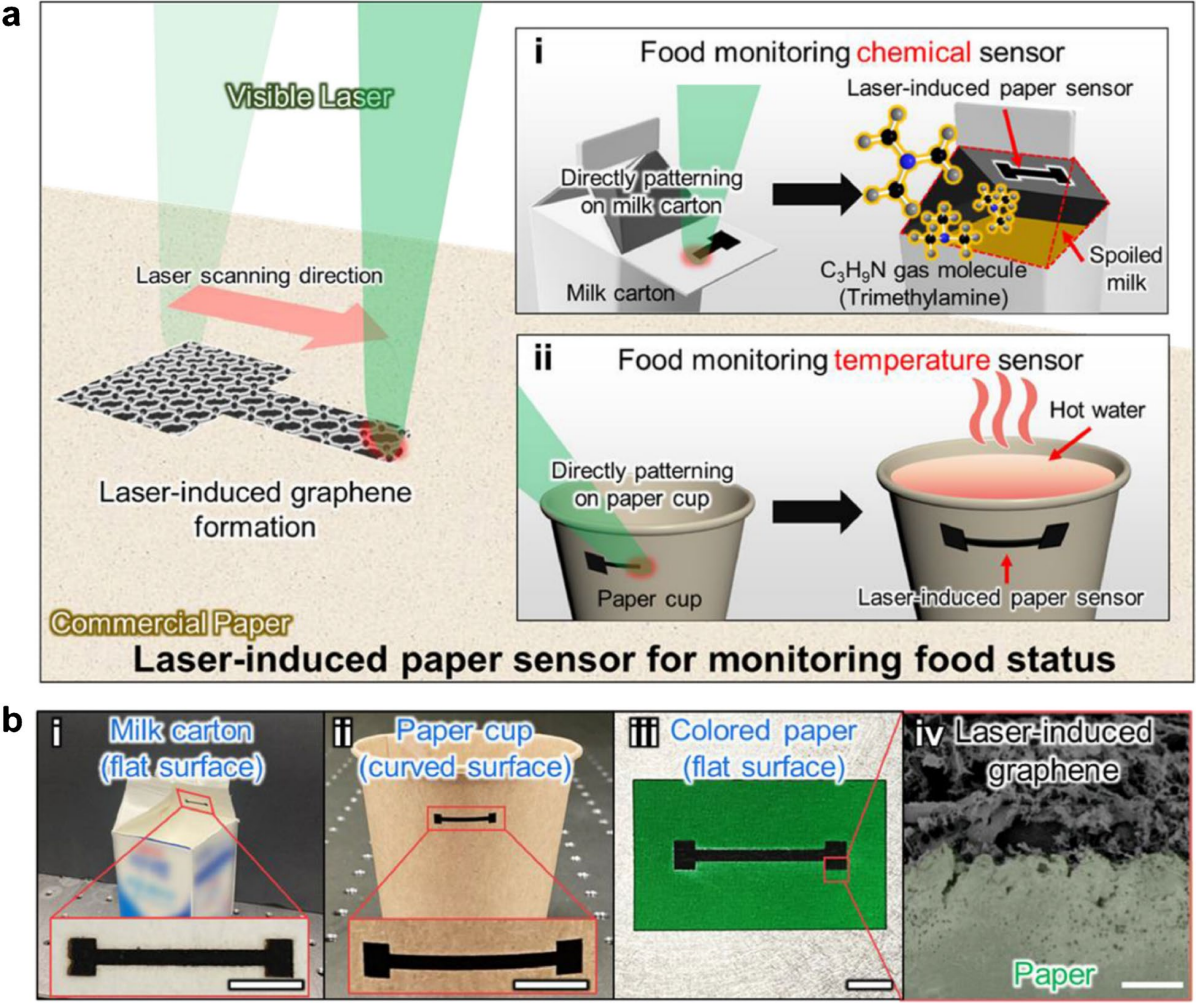
Schematic demonstration of LIPS fabrication. **a** Development of LIG on paper by laser induction. a-i) LIPS developed on the milk carton for food monitoring. a-ii) LIPS is designed on paper cups for the monitoring of food temperature. **b** Photograph of LIG fabricated on different materials. b-i) LIG fabricated on the milk carton. b-ii) LIG on paper cup substrate. b-iii) LIG on colored paper precursor. b-iv) A SEM image of LIG was fabricated on colored paper. Reproduced from Elsevier under copyright © 2022 Elsevier B.V. [7]

Jung et al. [7] designed smart paper flexible electronics by LIG to monitor food spoilage in real time. In this study, an LIG paper sensor was reported for wireless observing of food status through chemical- and thermo-sensing. Laser irradiation on paper transformed it into laser irradiated paper surface (LIPS) with sheet resistance 105 Ω sq^−1^. The porous structure of LIPS enabled the smart sensing of temperature and gas emissions from food samples. Laser induction at optimized conditions generated temperature and gas coefficient of resistance change 0.15%°C^−1^ and 0.0041% ppm^−1^. In addition, LIPS was fabricated on paper to monitor temperature and detect the decomposition of food by transferring data directly to mobile by wireless communication (Fig. 12). The designed LIPS sensor device can be used for commercial applications to monitor the status of food and dairy products in real time with greater fidelity [7].

**Fig. 13:**
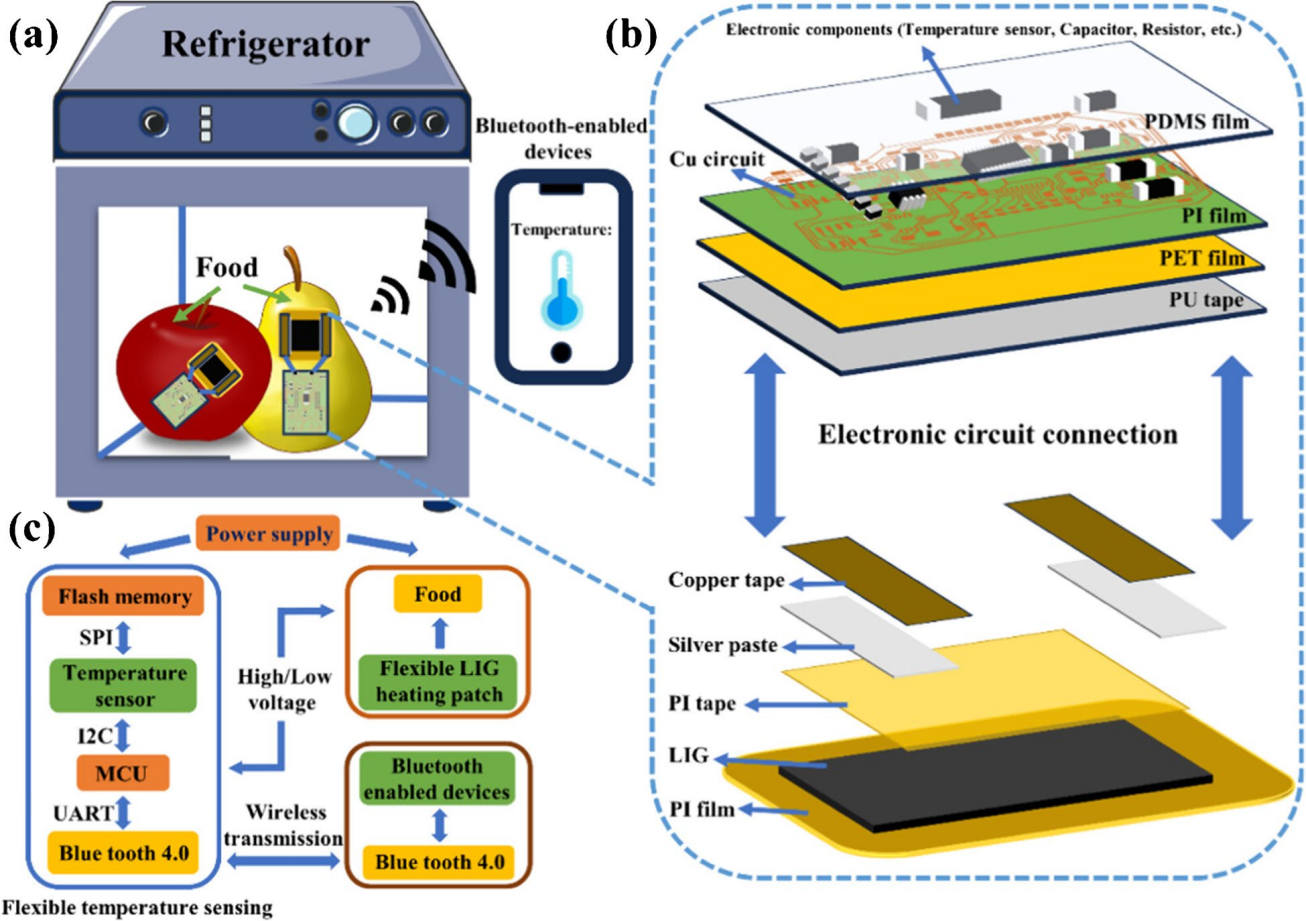
Design of flexible sensing and heating system based on frostbite resistance in fruit cold storage. **a** Schematic presentation of flexible sensing and heating system on fruits. **b** Hardware layout of flexible sensing and heating system. **c** Diagrammatical illustration of flexible sensing and heating system. Reproduced with permission from American Chemical Society under copyright CC BY 4.0 [172]

Another study was conducted by Chen et al. [172] to develop an LIG-based sensing system for the monitoring of food samples. The LIG-based flexible heating and sensing system was used to protect and store food in cold storage for long periods of time. The proposed system comprises two main parts: a flexible temperature sensor and a LIG heating component. Different electrical and structural attributes of the LIG heating component were studied to obtain the best performance. The designed LIG heating system indicated the best performance at a power of 12.5% and a scanning speed of 120 mm/s. For the practical applications, an anti-frostbite test was performed on fruits at the output voltage of 3 V. It was observed that this process takes only 14–20 s to set the range, and obtained results indicated that the designed system can effectively resist frostbite and sense the temperature of fruits surfaces precisely (Fig. 13). Hence, this strategy has promising applications in reducing food spoilage [172].

**Fig. 14:**
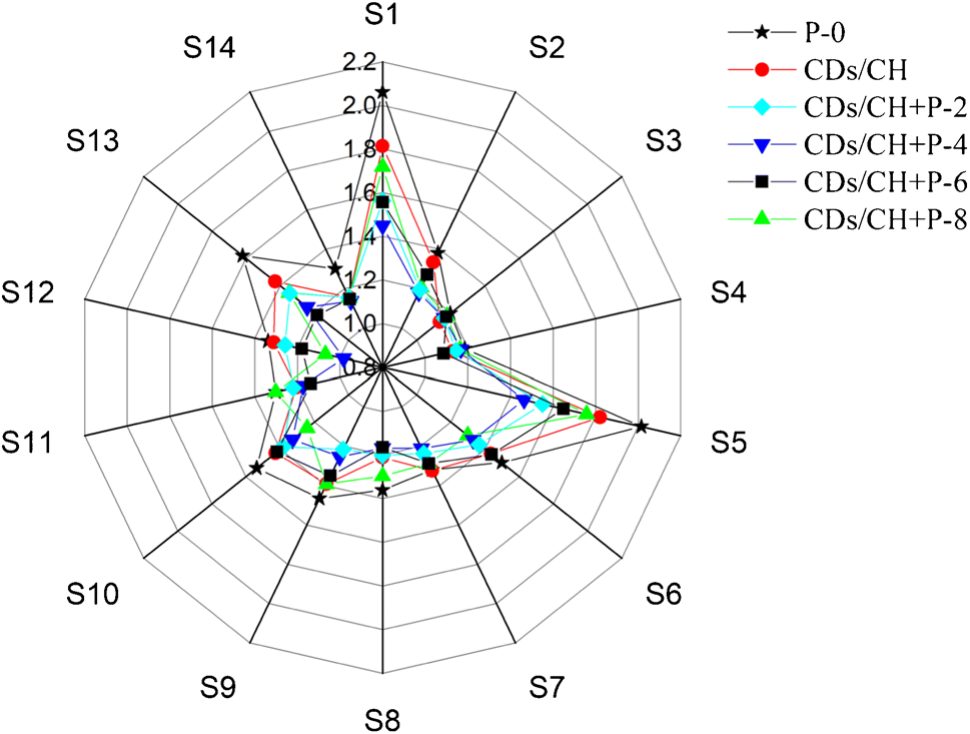
Spider web graph shows the monitoring of flavor variations of fresh-cut cucumber after 15 days with laser-induced microporous packaging (100 μm) and coating with chitosan carbon dot. Reproduced with permission from Springer Nature under Copyright @ 2021 [173]

Zhang et al. [173] developed a food preservation method based on laser-induced microporous packaging and coating with chitosan carbon dots. Modified atmosphere packaging (MAP) is extensively applied to preserve fresh food samples, including fruits and vegetables. The laser-induced MAP and carbon dot-chitosan coating effectively adjusted the gas composition during the fresh-cut cucumber storage period. The developed method supplied suitable oxygen and carbon dioxide concentrations of about 9.8% and 10.3%, respectively. The laser-induced microporous MAP (100 μm) coated with carbon dot-chitosan minimized the weight loss of about 4.1%, prevented flavor decomposition, and maintained it for 15th days in water distribution behavior (Fig. 14). Hence, this MAP method can be used effectively for the preservation and storage of fresh fruits and vegetables [173].

### Biogenic amines and food additives

Biogenic amines are nitrogenous compounds with significant toxicological effects associated with food safety, food quality, and waste. High levels of biogenic amines in food and dairy products can cause food poisoning. On the other hand, low levels of biogenic amines may cause food intolerance. Biogenic amines in food samples are generated from endogenous enzymatic activity or microbial contaminations, which direct the decarboxylation of amino acids. There are different threshold concentrations of biogenic amines in different countries [174–177]. Similarly, food additives are commonly used in the food and dairy industry, and their high levels have severe effects on human health. Therefore, the precise and accurate monitoring of food additives and biogenic amines is important to meet the limit suggested by food regulating authorities. LIG has been recommended to develop high-performance biosensors to detect biogenic amines and additives in food and dairy products [178–181].

**Fig. 15:**
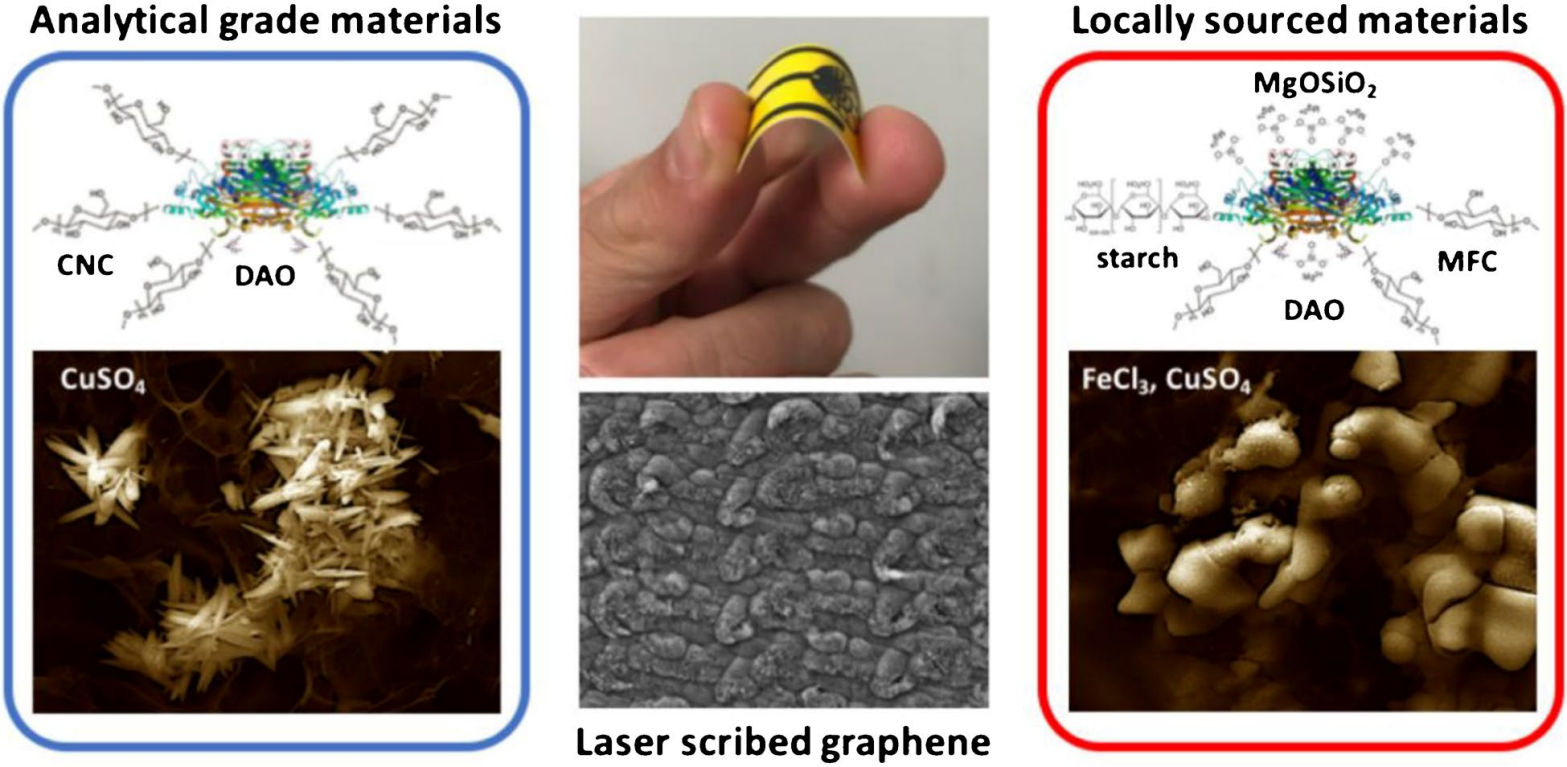
Schematic elaboration of a cost-effective disposable LSG biosensor for the sensing of biogenic amines in food samples by applying locally sourced materials and comparison was made with fabricated sensor of analytical grade materials. Reproduced with permission from MDPI under copyright CC BY 4.0 [150]

A study was conducted by Vanegas et al. [150] to develop an LIG biosensor for sensing biogenic amines (BA) in food products. In this study, the LIG electrode was produced from locally sourced substrates. For the fabrication of the biosensor, the LIG surface was modified with Cu microparticles and diamine oxidase. The designed amperometric LIG biosensor indicated promising performance with a lower limit of detection of 11.6 µM and a rapid response time of about 7.3 s. For practical applications, the fabricated LIG biosensor was used to detect total biogenic amine levels in fish food samples exposed to the fermentation process with lactic acid bacteria. This sensor indicated selectivity for BA even in complex food samples (Fig. 15). Hence, the developed sensor is highly suitable and can be used effectively to detect biogenic amines in food and dairy products. In addition, it can be used to monitor food samples while minimizing food waste and limiting foodborne poisoning [150].

Similarly, Salvia et al. [182] fabricated wireless self-driven LIG biosensors for the monitoring of biogenic amines. Conventional biosensors require complex electronic devices, which limit their broad-spectrum applications. However, the LIG strategy has a simple and cost-effective setup in which complex circuits are printed on polymeric substrates by laser induction. In this study, a zero-power self-regulated wireless LIG biosensor based on polyimide was developed to sense amine in food samples. The interdigitated sensor and communication antenna were fabricated purely by LIG induction, with non-uniform lasing attributes. The designed sensor was flexible and had the potential to detect triethylamine with less than 15% concentration and high readability of about 1.5 m due to resorting to the backscattering modulation framework [182].

**Fig. 16:**
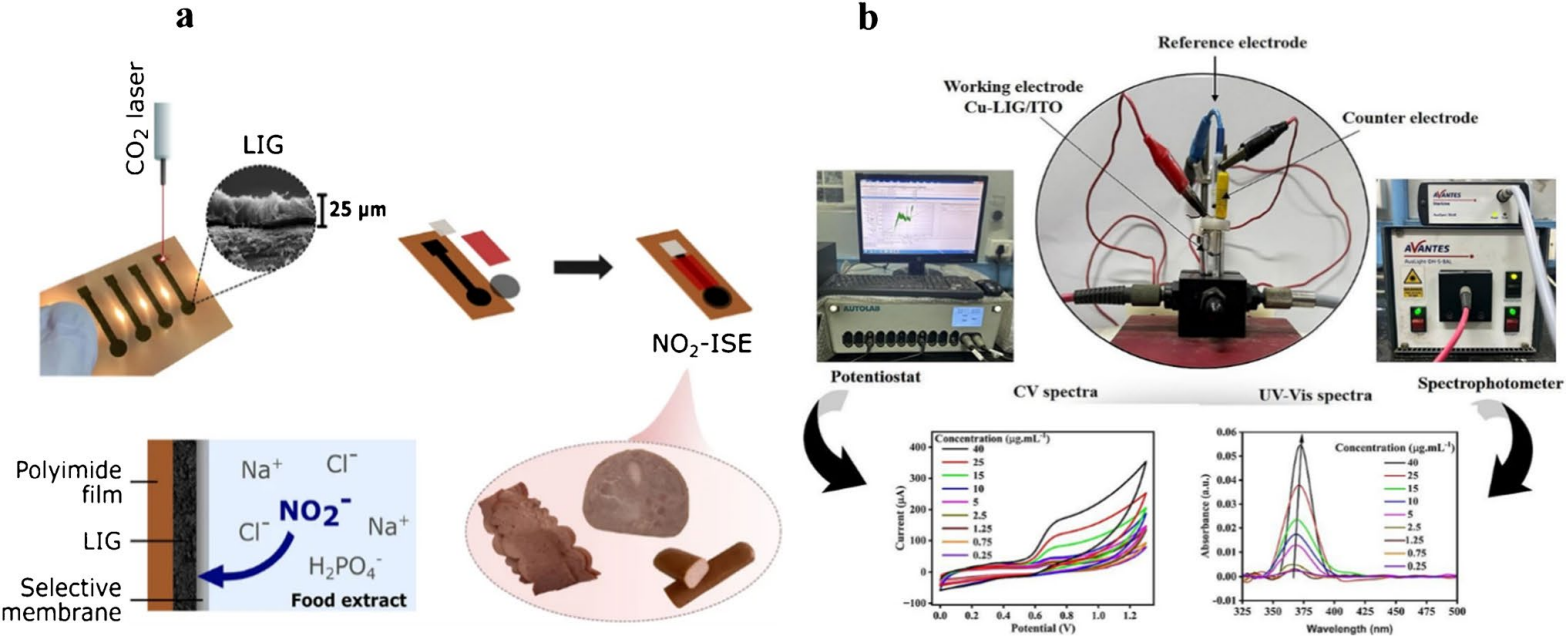
**a** Graphical explanation of LIG ion-selective electrode development for the sensing of nitrite in food samples. Reproduced with permission from Springer Nature under Copyright @ 2022 [151]. **b** Diagrammatical illustration for the fabrication of Cu/LIG modified electrode for the sensing of vanillin in food samples. Reproduced from Elsevier under copyright © 2024 Elsevier B.V. [81]

Soares et al. [151] designed ion-selective LIG electrodes for effectively sensing nitrite in food and dairy products. Nitrite is a commonly used food additive that adversely affects human health at high-level concentrations. Hence, the precise monitoring of nitrite levels in food and dairy products is highly mandatory. In this study, hydrophilic and hydrophobic LIG was fabricated, which transformed into an ion-selective biosensor to detect nitrite in food samples, and its performance was compared with the standard method (Griess method). The hydrophilic, ion-selective LIG electrode showed enhanced potential stability owing to a decrease in the water layer among the LIG electrode and ion-selective membrane based on nitrite polyvinyl chloride. The resulting designed sensor indicated a greater sensitivity of about 59.5 mV dec^−1^ and a low limit of detection of 0.3 ± 0.1 mg L^−1^ along with a broad linear concentration range between 10^−5^ and 10^−1^ M. In addition, the developed sensor was used successfully to monitor the nitrite level directly in food samples, including sausage, ham, and bacon (Fig. 16a). The above results indicated the versatility of ion-selective LIG electrodes for the development of biosensors with simple, cost-effective, and efficient sensing abilities. Hence, these sensors are highly promising in observing the nitrite concentration in food and dairy products effectively to ensure food regulatory compliance [151].

Another study was conducted by Gupta et al. [81] to develop Cu-LIG nanocomposites for the detection of vanillin in food samples. Vanillin is a food additive commonly used as a fragrant or flavoring compound in food and dairy products. However, excessive use of vanillin has deteriorating impacts on human health. Hence, precise and sensitive measurements of vanillin in food samples are highly recommended. In this study, vanillin was successfully monitored by applying the UV–Vis Spectro electrochemical technique. In this method, Cu-LIG nanocomposites were deposited on an indium tin oxide (ITO) electrode. The designed modified Cu-LIG/ITO electrode was applied to detect oxidized vanillin at particular wavelengths with cyclic voltammetry measurements at 0–1.3 V potential. The developed sensor indicated a linear concentration range between 0.25 and 40 μg/mL with an *R*^2^ value of about 0.993 and a lower limit of detection of approximately 0.14 μg/mL. In addition, the designed sensor was highly selective and indicated no response toward interfering substances like glucose, fructose, and ascorbic acid (vitamin C) in actual food samples (Fig. 16b). Hence, this technology can be used for the detection of additives in commercial food and dairy products [81].

### Antibiotics in food and dairy products

Antibiotics are the most commonly used secondary metabolites produced from particular fungal and bacterial strains. They have harmful impacts on human health and can play vital roles in the food, dairy, and medicine industries. Excessive use of antibiotics in animals leads to their accumulation in animal-derived foods like milk, meat, and dairy products. They put human health at risk by creating allergic reactions, antibiotic resistance, etc. [183–185]. The Food and Drug Administration (FDA) has set a certain threshold for the use of antibiotics in food and dairy products to save consumer health. Hence, the sensitive and precise detection of antibiotics in food and dairy products before consumption is highly mandatory to guarantee and guard human health [186–188].

**Fig. 17:**
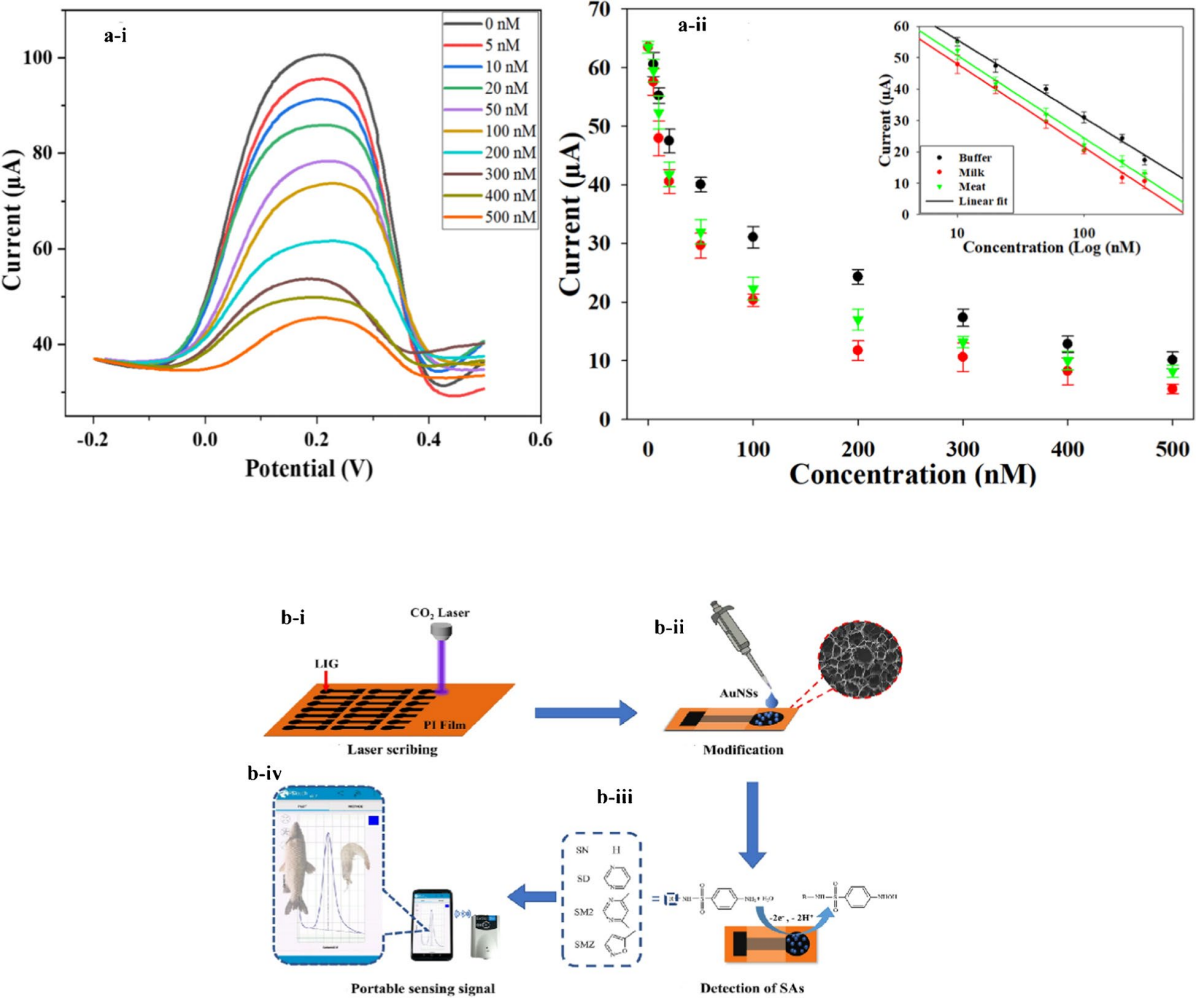
**a** Selectivity test of LIG electrode optimized by imprinted polymer-based biosensor for the sensing of tetracycline. a-i) The differential pulse voltammetry (DPV) curves against different concentrations of tetracycline. a-ii) Linear concentration range of the designed sensor. Reproduced with permission from MDPI under copyright CC BY 4.0 [2]. **b** Schematic demonstration for the development of LIG electrode modified by gold nanoparticles for the sensing of sulfonamide in shrimp food sample. Reproduced with permission from Springer Nature under Copyright @ 2022 [152]

A study was conducted by Abera et al. [2] to develop a LIG electrode optimized by imprinted polymer for the sensing of antibiotics (tetracycline) in food samples. In this work, three-electrode sensors were fabricated for the label-free sensing of tetracycline in food samples, including milk and meat. LIG electrodes modified with imprinted polymers and gold nanostructures were applied as biorecognition elements. LIG was developed on a polyimide sheet with a promising sheet resistance of about 17.27 ± 1.04 Ω/sq. The phenylenediamine monomer and tetracycline were molecularly imprinted on the LIG electrode. The designed sensor showed a very low limit of detection of about 0.85 nM in milk and 0.80 nM in meat samples. It also indicated a broad linear range concentration range between 10 and 300 nM (Fig. 17a). In addition, the developed sensor depicted good stability, reproducibility, and selectivity; hence, it can be used efficiently for the detection of antibiotics in food and dairy products [2].

Qiu et al. [152] designed a wireless flexible sensor based on a LIG electrode modified by gold nanoparticles for the sensing of sulfonamide in aquatic food products. In this study, LIG was developed on a polyimide sheet by laser induction, followed by the fabrication of gold nanoparticles on the surface of the LIG electrode. The developed sensor showed promising sensitivity towards sulfonamide with a linear concentration range between 0.4 and 100 μM and a lower limit of detection of about 0.035 μM. Furthermore, the fabricated sensor showed excellent recovery in the range of 96.04–105.00% in fish and shrimp samples. This sensor was integrated with a wireless Bluetooth device for real-life applications (Fig. 17b). The designed sensor indicated greater feasibility and practicability for on-site monitoring of sulfonamide in food samples to ensure food safety [152].

**Fig. 18:**
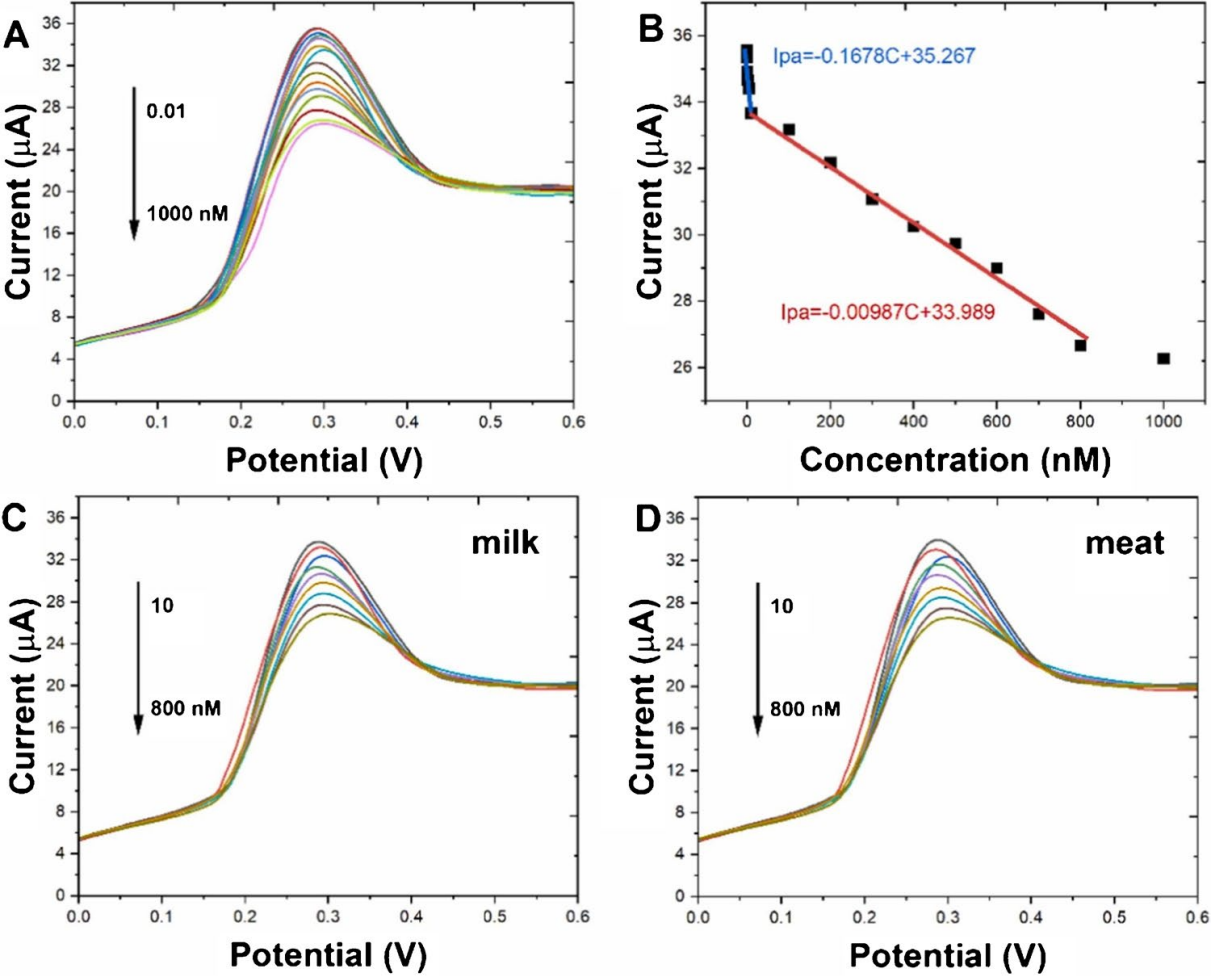
**A** DPV curves of AuNPs/LIG electrode against different concentrations of tigecycline. **B** Linear concentration curves of the designed sensor. **C** DPV curves of the fabricated sensor to detect the tigecycline in the milk sample. **D** DPV curves of the fabricated sensor to detect tigecycline in meat extract. Reproduced from Elsevier under copyright © 2024 Elsevier B.V. [153]

Similarly, another study conducted by Zheng et al. [153] developed a molecular imprinted sensor made of an LIG electrode for the monitoring of tigecycline. In this study, a LIG electrode modified gold nanoparticles (AuNPs) was developed to detect tigecycline in food samples. The molecular imprinted polymer-based AuNPs LIG electrode was electropolymerized by o-phenylenediamine and tigecycline substrate. The developed sensor exhibited a broad linear concentration range between 0.01 and 800 nM, with a lower limit of detection of about 0.003 nM. For real-life applications, this sensor was used to detect tigecycline in milk and meat food samples and indicated promising sensitivity with a lower limit of detection of about 0.15 nM and 0.21 nM, correspondingly (Fig. 18). In addition, the developed sensor depicted greater selectivity, stability, and reproducibility. These results demonstrated that designed sensors can be used effectively for the sensing of tigecycline in food and dairy products and have advantages over conventional methods by being facile, cost-effective, sensitive, and practical [153].

**Fig. 19:**
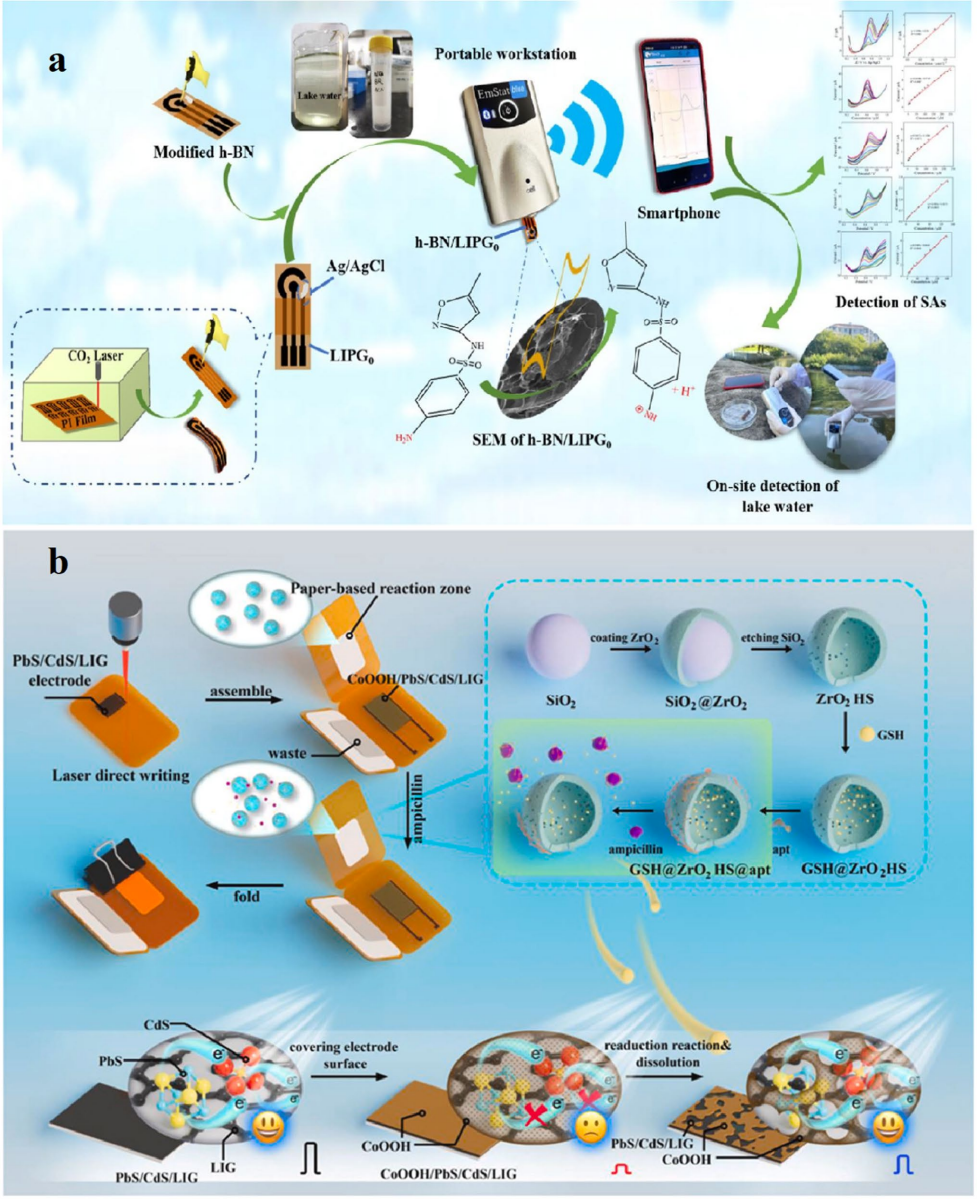
**a** Graphical demonstration of portable and flexible biosensor based on LIG for the sensitive monitoring of sulfonamide. Reproduced from Elsevier under copyright © 2022 Elsevier B.V. [154]. **b** Schematic illustration of paper-based biosensor device based on LIG/PbS/CdS electrode modified with CoOOH nanosheets for the sensing of ampicillin. Reproduced from Elsevier under copyright © 2022 Elsevier B.V. [155]

Zeng et al. [154] designed a portable LIG flexible sensor to monitor sulfonamides in food and water samples. In this work, the LIG electrode was modified with 2D hexagonal boron nitride, and the electrochemical performance was promising. The designed sensor was small, portable, flexible, and cost-effective to sense sulfamethoxazole at a low oxidation potential of about 0.56 V. This sensor showed a wide linear concentration range of about 0.5–362.5 µM, with a limit of detection 0.011 µM and excellent recovery range of about 97.5% in milk sample and 101.3% in lake water sample. Furthermore, the developed sensor was used to detect other sulfonamide drug products, including sulfanilamide, sulfapyridine, sulfadimidine, and sulfisoxazole (Fig. 19a). Hence, this sensor can be used for rapid and on-site monitoring of antibiotics in food and water samples to ensure food and environmental safety issues [154].

Qiu et al. [155] designed a portable, flexible, paper-based biosensor device composed of LIG/PbS/CdS electrodes modified with CoOOH nanosheets to monitor ampicillin antibiotics. In this study, ampicillin was integrated onto paper in a reaction chamber as a biogate aptamer to recognize a particular analyte. After the interaction with the target analyte, glutathione was released into the reaction chamber, and the etching of CoOOH nanosheets exposed the modified electrode (LIG/PbS/CdS) to generate a photocurrent signal. Paper-based designed sensor indicated a wide linear concentration range of about 5.0–2 × 10^4^ pM and lower limits of detection of about 1.36 pM. Furthermore, the developed sensor was used for real-time applications to sense ampicillin in milk and water samples and showed promising selectivity, excellent stability, and a higher percentage of reproducibility (Fig. 19b). These results demonstrated its potential applications in food sample analysis and environmental safety monitoring [155].

### Chemical contaminants and pesticides

The presence of chemical contaminants and pesticides in food and beverages has deteriorating impacts on human and animal health. Cost-effective detection of chemical contaminants and pesticides in food and dairy products is highly mandatory to ensure the quality and safety of food samples [189–192]. Hence, it is recommended that flexible, portable, consumer-friendly, and quantitative sensor devices be developed to monitor pesticides and chemical contaminants in food samples and guarantee food safety. Herein, LIG-based flexible sensors are suggested for sensing contaminants, and pesticides/insecticides in food samples [193–196].

A study was conducted by Mostaccio et al. [197] to develop a self-powered wireless LIG biosensor for the monitoring of triethylamine. In this study, an LIG-based self-powered biosensor device was constructed, integrated with an antenna and an interdigitated capacitor fabricated by laser induction to optimize the radiation performance and indicate the signal on the sensor device. The designed sensor is portable and can be studied from about 1.6 m via radio frequency identification (RFID). For the sensing applications, the sensor was coated with a chemical interactive material that was compatible to interact with triethylamine compounds. In addition, integrating developed sensors with machine learning algorithms made it possible to detect various concentrations of amine between 10 and 45%. These results suggested that developed sensors can be used effectively to monitor volatile organic compounds, pesticides, and insecticides in food and dairy products [197].

**Fig. 20:**
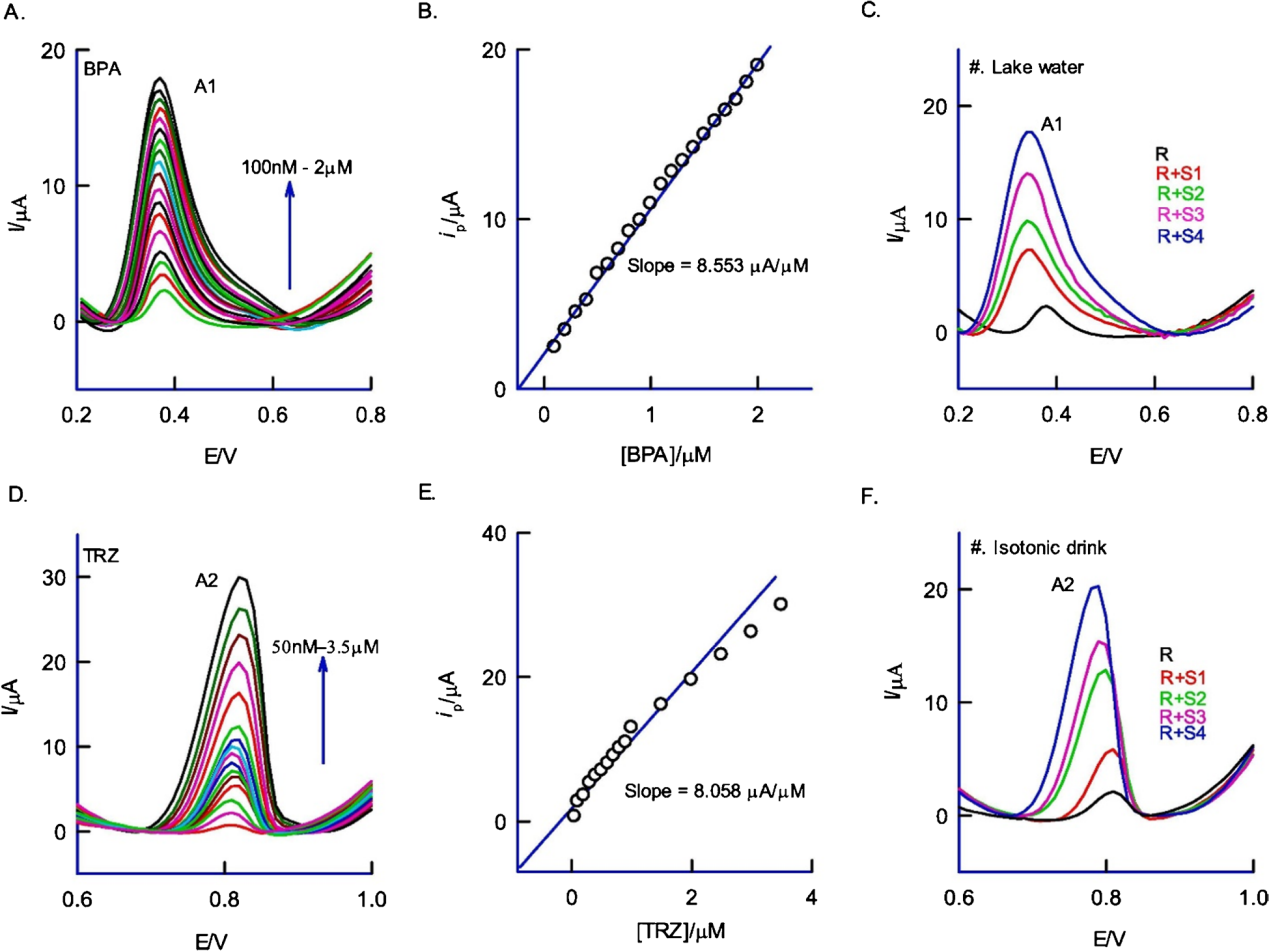
**A** Adsorptive stripping differential pulse voltammetry (AdsDPV) curves of fabricated LIG electrode-based biosensor against different concentrations of bisphenol A. **B** Correlation between peak current and bisphenol A concentration. **C** AdsDPV curves for real water sample analysis by using the standard addition method. **D** AdsDPV curves of the designed sensor against different concentrations of tartrazine. **E** Correlation between peak current and tartrazine concentration. **F** AdsDPV curves of the fabricated sensor for the monitoring of tartrazine in real isotonic drink sample by applying standard addition approach. Reproduced from Elsevier under copyright © 2024 Elsevier B.V. [198]

Similarly, another study was conducted by Vignesh et al. [156] to design a LIG electrode modified by Ag-La(OH)_3_@Dy_2_O_3_ composite for sensing bisphenol A and tartrazine in food samples. The modified LIG electrode showed an enhanced electron transfer rate and promising electrochemical sensing activity for bisphenol A and tartrazine with a lower limit of detection of 9.2 nM and 0.96 nM respectively. In addition, the designed sensor was used for real-life applications to monitor organic contaminants, including bisphenol A and tartrazine, in food samples (Fig. 20). Hence, a designed sensor is suggested for the real-time monitoring of organic contaminants in food and dairy products [198].

**Fig. 21:**
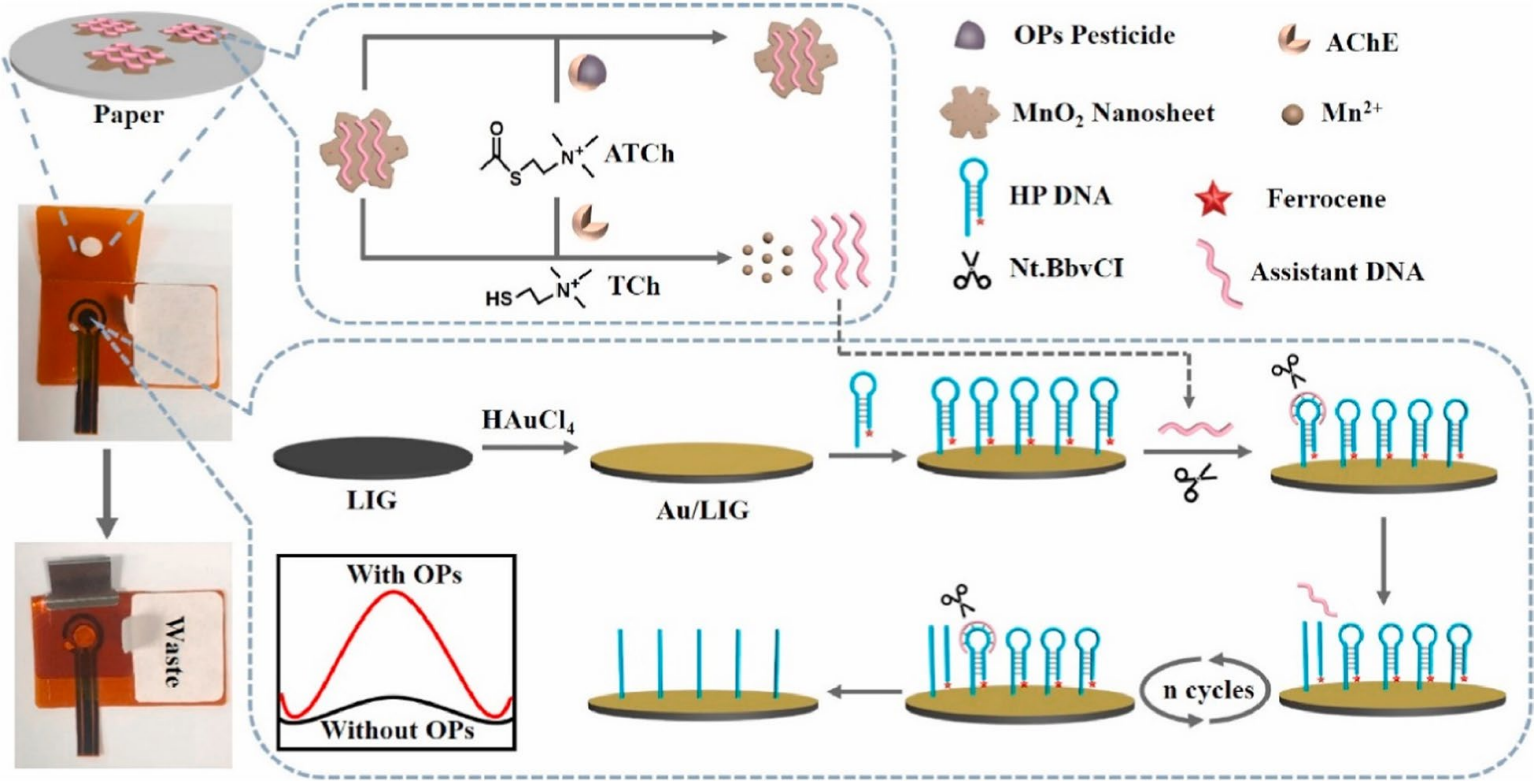
Graphical demonstration of proposed sensing mechanism of designed LIG and MnO_2_ linked DNA amplification-based sensor for the sensing of pesticides in food samples. Reproduced from Elsevier under copyright © 2021 Elsevier B.V. [157]

Liu et al. [157] developed a portable, flexible LIG and MnO_2_-linked DNA amplification-based sensor for the sensing of pesticides in food samples. Herein, the biosensor was developed by a LIG electrode patterned on polyimide foil and modified by MnO_2_ nanosheets deposited on paper with organophosphorus (OPs) substrates. The sensing mechanism of this sensor was based on acetylcholinesterase-initiated disintegration of MnO_2_ to release DNA signal to trigger enzyme-mediated amplification. The enzymatic activity of the acetylcholinesterase enzyme was inhibited in the presence of OPs and, resulting in the cleavage of the probe on LIG modified electrode, was not initiated. The generated electrochemical signals indicated the presence of OPs in food samples. Furthermore, the developed sensor indicated a wide linear concentration range between 3 and 400 ng/mL, with a limit of detection of about 1.2 ng/mL (Fig. 21). For the real-time application, this sensor was used to monitor the OPs levels in vegetable samples, and it showed excellent sensitivity. Hence, the developed sensor device can be used to monitor food quality and ensure environmental safety [157].

**Fig. 22:**
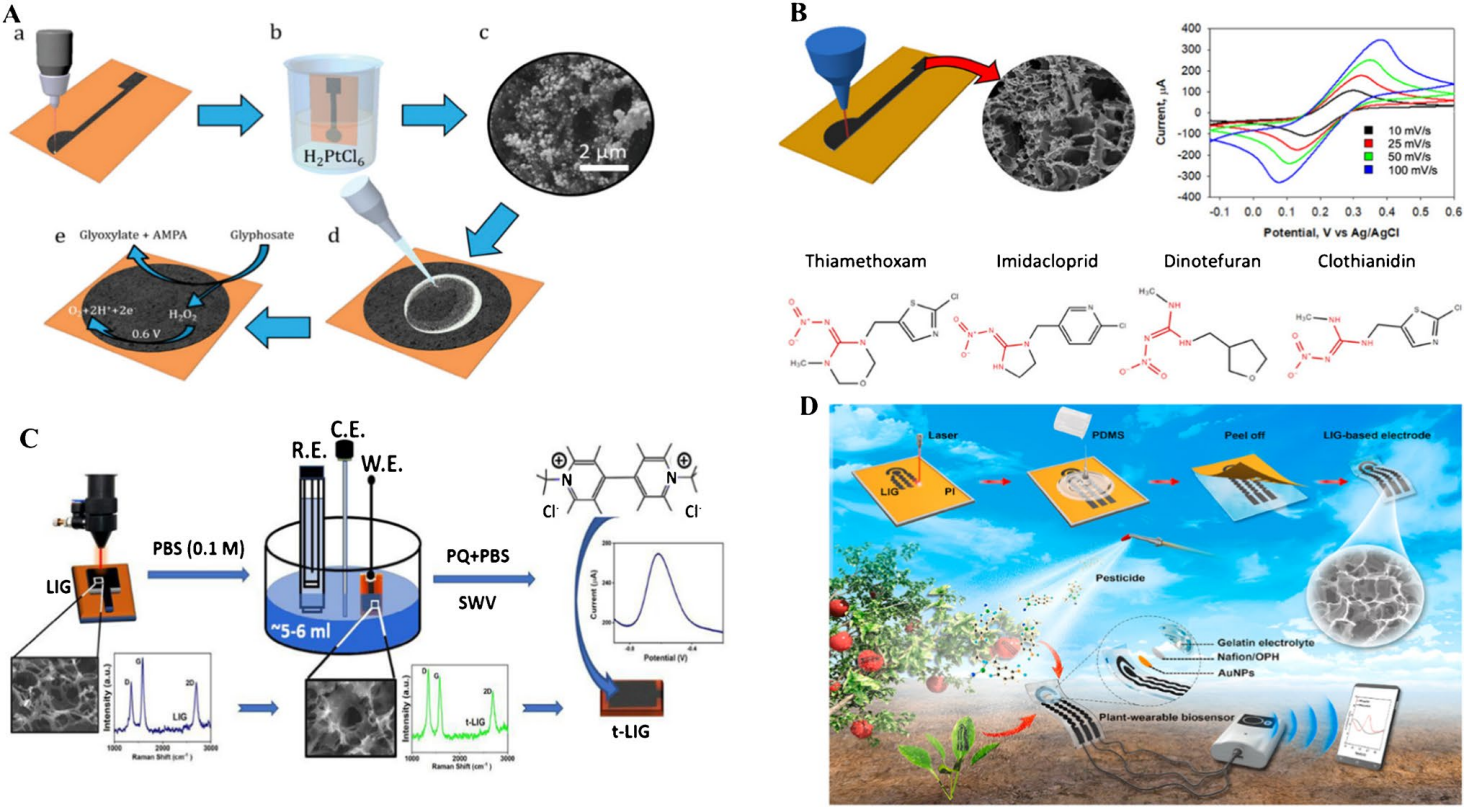
**A** Demonstration of glyphosate LIG sensor development and sensing mechanism. a) LIG patterning on polyimide (PI) sheet by laser induction. b) Platinum deposition at potential −0.5 V against Ag/AgCl. c) SEM image of platinum-modified LIG electrode. d) Drop casting of glutaraldehyde, flavin adenine dinucleotide and glycine oxidase. e) Sensing mechanism of glyphosate via electrochemical oxidation at potential 0.6 V. Reproduced with permission from WILEY under copyright CC BY 4.0 [158]. B Schematic illustration of neonicotinoids sensing by using LIG sensor. Reproduced with permission under copyright CC BY 4.0 [199]. C LIG sensor for the electrochemical sensing of paraquat in water. Reproduced with permission under copyright CC BY 4.0 [200]. D Explanation of 3D porous LIG flexible plant sensor for pesticide monitoring. Reproduced from Elsevier under copyright © 2020 Elsevier B.V [201]

Similarly, Johnson et al. [158] conducted a study to develop LIG derived biosensor for monitoring the herbicide glyphosate. Glyphosate is a commonly used herbicide and has a harmful impact on farmers and crops. In this study, an LIG sensor was constructed to rapidly sense glyphosate and ensure the safety of farmers, crops, and water. The glycine oxidase enzyme was immobilized on the surface of the LIG electrode modified with platinum to monitor the glyphosate. The developed sensor exhibited a broad linear concentration range of 10 to 260 µm and a lower limit of detection of about 3.03 µm with a sensitivity of approximately 0.991 nA µm^−1^. Furthermore, the designed sensor showed excellent selectivity in the presence of interfering herbicides as well as insecticides (Fig. 22). This sensor was used to monitor glyphosate in real water and crop residue samples. These results demonstrated the potential of a designed sensor to be applied for sensing of herbicides in food samples [158].

Commonly used pesticides like neonicotinoids cause significant risks to pollinators, ecosystems, and human health. However, there is still a lack of real-time tools to detect pesticide residues in the aquatic ecosystem. Johnson et al. design an advanced electrochemical sensor system, based on LIG to cover up these critical gaps by quick, label free sensing of four major neonicotinoids including clothianidin (CLO), imidacloprid (IMD), thiamethoxam (TMX), and dinotefuran (DNT), showing low limit detection and fast response time without chemical functionalization. These LIG sensors are fabricated using a scalable and mask-free laser procedure, revealing high selectivity and effective differentiation of neonicotinoids from common pesticides. This cost-effective platform of LIG sensors meets the criteria for monitoring pesticide traces in laboratories and will play a significant role in future field implementable applications. This step is crucial for protecting the environment from detrimental chemicals and pesticides [199].

The increasing concern over **pesticide and herbicide residues, particularly highly toxic and persistent paraquat (PQ)**, is causing severe environmental and public health concerns, necessitating advanced detection approaches. Traditional techniques, such as UV–vis spectroscopy, chromatography, and fluorescence titration, are often expensive, complex, labor-intensive, and limited to lab experiments. That’s why here is the illustration of flexible, field deployable electrochemical sensors based on laser induced graphene, specifically square-wave voltammetry (SWV), which are sensitive, fast, and an effective tool for the first time detection of PQ. Optimization and surface characterization of LIG through FE-SEM and Raman spectroscopy improved its electrochemical performance, porous structure, and hydrophilicity. The selectivity and sensitivity of LIG-based sensors in order to detect PQ can be optimized by optimizing parameters like pH and deposition potential. Real-world application of flexible LIG-based sensors was endorsed for the first time in the tap water sample, indicating the significant potential for the quick, real-time detection of pesticides like PQ in Laboratories and fields [200].

Similarly, for the detection of organophosphorus **pesticide,** a flexible electrochemical sensor using laser-induced graphene was designed that is patterned on polyimide to generate three three-electrode system, with a combination of **pralidoxime (PAM)** and **CeO**_**2**_** for enhancement of electrocatalysis. Electrochemical properties, optimized fabrication parameters, portable, cost-effective, highly sensitive, and stable LIG sensors demonstrated outstanding detection of pesticides. Its excellent sensitivity, repeatability, stability, and anti-interference make it appropriate for the detection of organophosphorus pesticides both in field and lab-based monitoring **[201]**.**

The plant-wearable biosensors fabricated through LIG and transferred through polydimethylsiloxane (PDMS), modified with organophosphorus hydrolase (OPH), gold nanoparticles (AuNPs), and a biocompatible semisolid electrolyte, can be used for fast, in situ, selective detection of methyl parathion, providing real-time data wirelessly transmitted to a smartphone for further electrochemical analysis. This innovative approach offers a significant tool for onsite monitoring of pesticide residues in the laboratory and field, thus promoting the smart and precision farming system [202].

Studies show the development of a **biomimetic fern leaf pesticide collection patch** and an **electrochemical LIG biosensor** to generate a “collect-and-sense” system for rapid pesticide spray monitoring in the field. In this setup, the LIG patch gathers pesticide spray and electrochemical sensors integrated with platinum nanoparticles and glycine oxidase, which detects glyphosate precisely. This portable, scalable system enables remote analysis with 97% accuracy compared to commercial systems. It is also helpful for onsite, rapid pesticide monitoring in labs and fields, supporting precision agriculture, and lessening environmental impacts [203].

**Fig. 23:**
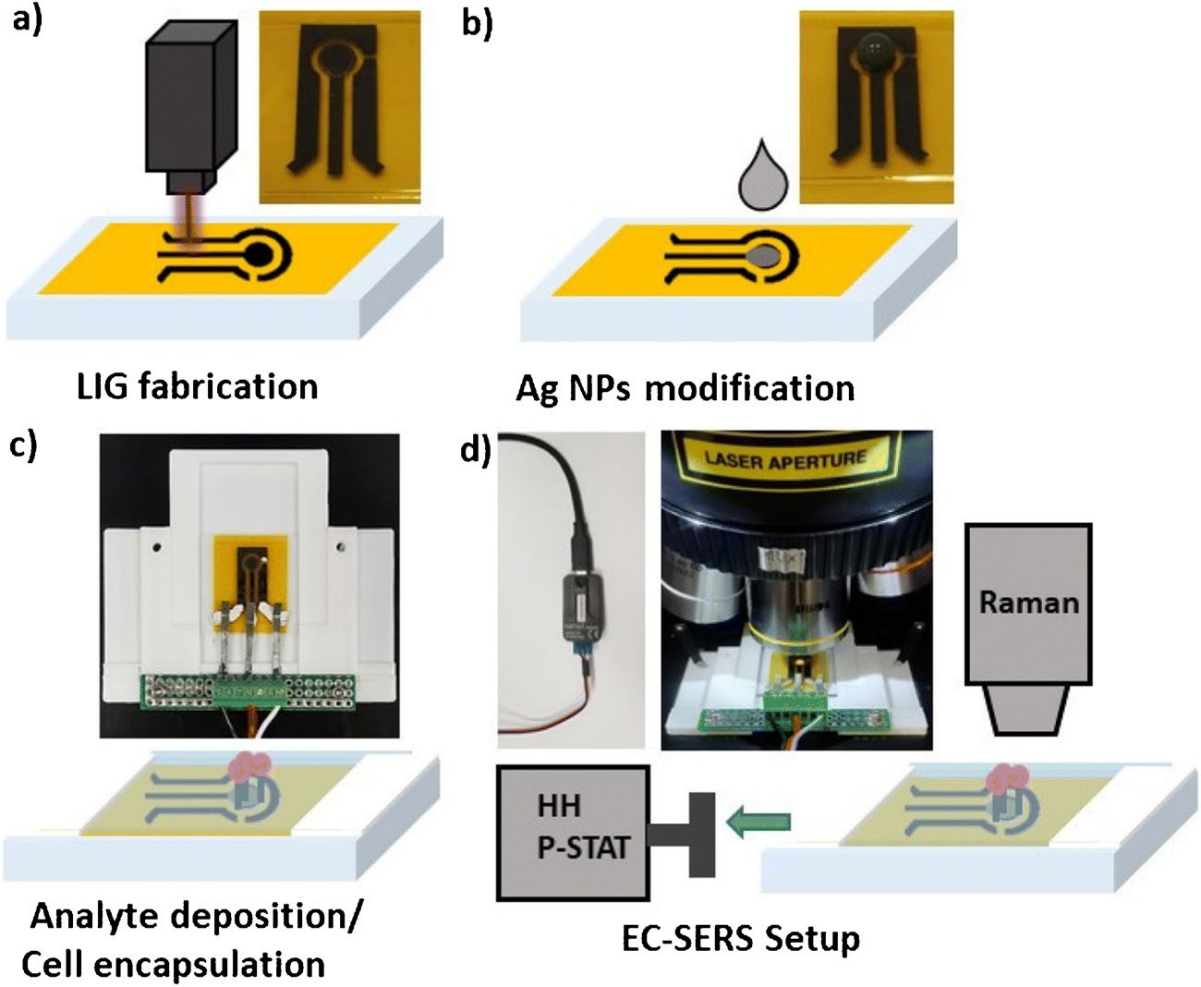
**a** Schematic elaboration of LIG electrode fabrication by laser induction on polyimide sheet. **b** LIG surface modification by silver nanoparticles (AgNPs). **c** Immobilization of analyte and cell encapsulation on LIG electrode surface. **d** EC-SERS monitoring setup. “Silver nanoparticles–laser-induced graphene. Reproduced with permission from Royal Society of Chemistry under copyright CC BY-NC 3.0 [202]

Mu et al. [202] developed an LIG hybrid electrode modified with silver nanoparticles (AgNPs) for the detection of food contaminants by electrochemical sensing based on surface-enhanced Raman spectroscopy (SERS). Herein, the LIG/AgNPs modified electrode showed excellent electrocatalytic activity by integrating potentiostat with Raman spectroscopy that enabled the observation SERS spectra against particular analytes. The developed sensor was used to sense 4-aminobenzenethiol (4-ABT), melamine, a food contaminant of milk, and antibiotics in a river water sample (Fig. 23). Furthermore, this sensor showed excellent sensitivity and a broad linear concentration range. Simple and cost-effective fabrication of developed sensors make it a promising candidate for large-scale monitoring of food samples and environmental analysis [202].

## Current challenges and future outlook

Laser-induced graphene (LIG) has gained huge attention in biosensing applications owing to its unique physicochemical and rheological properties. LIG biosensors are promising devices in food and dairy industry applications. They have low toxicity, excellent biocompatibility, and highly sensitive properties, which make them favorable candidates for biosensing [201, 203]. In addition, LIG biosensors integrated with artificial intelligence, machine learning, self-powered and wireless communication systems enable them for advanced sensing applications in food and dairy industries [204, 205]. Furthermore, LIG sensors based on natural substrate materials, including leaves, wood, and polymers, have higher biocompatibility and, hence, can be used effectively to fabricate green electronics biosensor devices. Although LIG biosensors have gained significant advancement still there are certain limitations and challenges that need to be addressed for practical applications [206, 207].

The selection of a suitable material is critical for developing good-quality LIG. There are a lot of structural defects that are mandatory for the practical applications of LIG biosensors. Sometimes, amorphous carbon is produced along with LIG during the laser induction process. Therefore, it is challenging to develop LIG sheets with uniform arrangement [55, 208]. However, defects and impurities can impact the physiochemical attributes by providing greater electrocatalytic activity, transport kinetics, and higher energy states. Another challenge is to reduce inevitable impure products like amorphous carbon during fabrication. Impurities from natural materials have a greater influence on the performance of LIG biosensors. Hence, suitable purification methods must be developed, which should be simple and cost-effective. Future research will facilitate comprehending associations among porous structures, substrate chemistry, and laser induction conditions to gain particular sensing properties [100, 209].

LIG fabrication mechanisms need to be optimized to control defects in LIG development. Further research and deep understanding would direct us to develop LIG from precursor substrate with elaborate doping elements, which can change the electronic configuration of graphene. These doping elements enhance LIG’s sensing abilities. Generally, LIG is integrated with other materials for biosensing. To optimize the sensitivity of the designed LIG biosensor, it should be integrated with a material that has a higher surface area and tunable energy bandgap. For this purpose, LIG with 3D and porous structures besides conductive properties is suggested for biosensor fabrications. Furthermore, large-scale production of LIG biosensors with sensitive, reliable, and stable performances is still challenging. To overcome these limitations, the roll-to-roll fabrication method is suggested for automated and commercial-scale production of LIG-based biosensors [133, 210, 211].

LIG biosensors must be compatible with flexible wearable electronic devices for practical applications. The consistency of sensing abilities, when biosensor devices are damaged, is a major challenge. To cope with this challenge, it is recommended that biosensors should be developed, such as a biosensor device small enough to minimize the deformation as much as possible. Furthermore, the integration between the LIG sheet and the conducting substrate layer is not too much and can be separated from each other by mild scratching. In addition, flexible biosensor devices that often undergo bending issues must be addressed during the fabrication process. Briefly, substrate transfer, nanocomposite for LIG fabrications, and device structure and configurations are highly promising strategies to attain desirable biosensors for food and dairy applications [212–214].

Safety is a great challenge for biosensors applied in food and dairy applications and puts risk to human health due to inhalation of graphene. The cytotoxicity studies of graphene and graphene-derived materials indicated varying levels of toxicity to model animals, like morphological disorders to zebrafish, and inflammation was observed in mice and rats as well. Surprisingly, LIG showed no toxicity to model animal zebrafish even at higher concentration [215–217]. However, it is suggested to do further research to ensure the cytotoxic effects of LIG and possible prevention for the leakage of graphene. This can be achieved by coating or cross-linking nanocomposites with LIG, which stops graphene detaching from LIG and maintains its porous structure as well [218, 219].

To date, most LIG biosensing is based on electrochemical methods, which have certain limitations like less sensitivity and selectivity. To combat these challenges, fluorescence, surface-enhanced Raman spectroscopy, and quartz crystal microbalance-based LIG biosensors are suggested for optimized biosensing applications [65, 96, 220]. Furthermore, it is recommended that multimodal LIG biosensors be developed for practical usage owing to their simple fabrication mechanism, direct laser writing process, and surface modifications for multiplexing. Moreover, in the future, LIG biosensors should be embedded with different flexible devices and self-regulated triboelectric nanogenerators (TENGs) for automated sensing applications. The performance of LIG biosensors, including stability, biocompatibility, conductivity, and electrochemical activities, can be enhanced further by controlling the surface morphology, porous structure, and functionalization. LIG doping with suitable dopant elements is a promising strategy to develop more precise and robust biosensor devices for advanced biosensing applications [55, 100, 221, 222].

The long-term structural and functional stability of LIG electrodes, particularly under different storage conditions, is still challenging. Similarly, analyte selectivity in the presence of structural analogue molecules in complex food matrices remains a vital challenge. These limitations need to be addressed by material engineering and surface modification strategies. Furthermore, concerns are present regarding the safety and biocompatibility of LIG, especially the residual dopants. Hence, it is suggested that green synthesis approaches be used, surface coating with biocompatible materials, and post-synthesis treatment to minimize potential toxicity. To ensure safety, clinical trials and pilot studies are required for validation. Although LIG development is cost-effective than other graphene synthesis approaches, high-quality and industrial-scale production remains challenging [223].

Physical parameters such as temperature, humidity, and pressure hinder the precision and accuracy of LIG sensors. Hence, it is mandatory to minimize interference among these physical parameters to obtain reliability in multimodal biosensors. This can be achieved through complicated signal processing machine algorithms, AI integration, and optimizations in sensor design. In short, it can be summarized that deep research and continued efforts are demanded in LIG direct fabrications with desired features, which will stimulate rapid advancements in the biosensors field [100, 223]. LIG-based sensors have emerged as a strong diagnostic apparatus in the real-life transformation to the field and commercial level because of their unique characteristics like a low-cost fabrication process, reconfigurable surface nature, and electroanalytical performance. They are highly compatible with malleable substrates and additive manufacturing that includes packaging of food, polymer layers, and marking of laser on paper, and they are supportive of incorporation into portable, wearable, and consumable strategies. Wireless data transmission, smartphone-based statistics, and self-powered procedure could be possible by launching advance, in situ monitoring systems for food safety. LIG biosensors have fascinating resolution for decentralized food quality assurance, scalable production, and environmental sustainability, specifically where there are limited resources for monitoring supply chains [37].

In the current era, the advancement in machine learning (ML) and AI has played an innovative role in optimizing LIG-based biosensors like the use of the Bayesian optimization approach to systematically tune the laser parameters, including speed, power, wavelength, pulse frequency, and exposure time, to enhance the quality and electroanalytical performance of LIG electrodes. ML-driven models minimize experimental trials and improve the reproducibility of LIG development. The ML algorithms, such as support vector machine and artificial neural networks, facilitate determining material properties and LIG sensor performance based on fabrication methods and environmental exposures. Hence, the incorporation of ML and AI into LIG synthesis will enhance the precision, sensitivity, and reproducibility of designed sensors [224, 225]. Table 3 summarizes how LIG sensors are applied to detect food decay and smart packing. These flexible sensors, due to wireless communication, monitor gas emission, temperature, microbial activity, and real-time, onsite status tracking of food packaging. This table also presents the emerging potential of LIG sensors in innovative food safety systems.

**Table 3:** Advantages, disadvantages, and requirements for LIG-based-biosensors in the food and dairy industry

Advantages	Disadvantages/limitations	References
Ease of use Fast response Higher sensitivity Higher selectivity Cost-effective Wide detection range Wireless communication Point of care devices Environmentally friendly	Complex matrix Changing metabolites Crosslinked constituents Multi-component analysis Cleaning and safety Biocompatibility Mechanical stability Calibration	[37, 43, 226]
Requirements		
Food safety Fast response and recovery time Reliability and reproducibility Improved limit of detection Integration with AI, IoT, and machine learning Self-regulated Self-powered Flexible and smaller size Approved by regulatory authorities Low maintenance Greater sustainability		[5, 224, 225]

## Conclusion

LIG has been utilized extensively in developing advanced biosensors. The cost-effectiveness, adjustable surface chemistry, eco-friendliness, and ease of fabrication make LIG a suitable candidate. There are extensive details about various approaches to fabricating LIG through several laser systems, precursor materials, and amenable surface approaches, helping the LIG-biosensors to boost their activity. Several fundamental sensing approaches, reinforcing the analytical performance of the LIG, such as capacitive, piezoelectric, electrochemical, piezoresistive, and FET-based techniques, are explained extensively in this review. Additionally, there is a focus on the wide role of LIG in the food and dairy industry, for the sensitive, on-site, and precise monitoring of food pathogens, antibiotics, additives, and deterioration indicators. As there is higher demand for the maintenance, safety, and quality of food, LIG biosensors are considered remarkable tools because of distinctive features like compatibility, miniaturization potential, and flexibility with digital packaging and IoT. Optimization and surface functionalization of LIG electrodes with suitable doping elements enhance the sensitivity of the designed sensors. Various LIG-derived high-performance biosensor devices have been developed to date. Integrating LIG-based biosensors with AI, IoT, MIP, and machine learning can yield advanced biosensors for various applications.

## Data Availability

No datasets were generated or analysed during the current study.
